# Potential of Native Brazilian Fruits in Modulating Oxidative Stress and Inflammation: A Focused Review

**DOI:** 10.3390/antiox15060677

**Published:** 2026-05-28

**Authors:** Maria Carolina Zsigovics Alfino, Geni Rodrigues Sampaio, Adriano Costa de Camargo, Elizabeth Aparecida Ferraz da Silva Torres

**Affiliations:** 1 Department of Nutrition, School of Public Health, University of Sao Paulo, 715 Doutor Arnaldo Ave, Sao Paulo 01246-904, Brazil; carolalfino@usp.br (M.C.Z.A.); 2 Instituto de Ciencias Aplicadas, Universidad Autónoma de Chile, Santiago 7500910, Chile

**Keywords:** inflammation, phenolic compounds, diet, native Brazilian fruits, public health

## Abstract

Chronic non-communicable diseases are closely linked to low-grade inflammation and oxidative stress. Native Brazilian fruits, rich in bioactive compounds such as polyphenols and carotenoids, have recognized antioxidant, anti-inflammatory, and neuroprotective properties. Mechanisms of action included inhibition of NF-κB signaling, downregulation of pro-inflammatory cytokines, modulation of oxidative biomarkers, and improvement of metabolic parameters. Several studies demonstrate protective effects against insulin resistance, hepatic steatosis, cardiovascular alterations, and neuroinflammation, alongside modulation of gut microbiota. Most evidence is from animal or cell models, with scarce clinical trials, limiting translational applicability. Overall, native Brazilian fruits represent promising dietary sources of bioactive compounds that may contribute to the modulation of oxidative stress and chronic inflammation. Challenges include variability in phytochemical content due to species, maturity, processing, a lack of standardized extraction and dosage protocols, and limited bioavailability data. Although preclinical findings are robust, further controlled human trials are necessary to confirm their efficacy and clarify their role in the clinical management of inflammatory and neurodegenerative diseases.

## 1. Introduction

Inflammation is an essential biological response to harmful stimuli, such as infections, injuries, and toxins, playing a critical role in maintaining human homeostasis and immune defense [1]. However, when sustained at low levels over time, chronic inflammation is associated with the development of numerous non-communicable diseases, including cardiovascular diseases, type 2 diabetes, and neurodegenerative disorders [2]. Neuroinflammation refers to a specialized inflammatory response within the central nervous system (CNS), primarily mediated by microglial cells and astrocytes, which can be triggered by genetic, environmental, and nutritional factors [3,4]. Persistent activation of microglia and astrocytes promotes the release of pro-inflammatory cytokines, compromising neuroplasticity and neurotransmission, and contributes to the pathogenesis of neurodegenerative and psychiatric disorders, such as Alzheimer’s disease, Parkinson’s disease, and depression [4,5].

In this context, dietary interventions are a promising strategy to modulate oxidative stress as well as systemic and neuroinflammatory responses. Phytochemicals, including polyphenols, flavonoids, anthocyanins, and carotenoids, are bioactive compounds found in plant foods that exhibit significant antioxidant and anti-inflammatory properties. “Anti-inflammatory” refers to the ability of a compound to reduce the production or activity of pro-inflammatory mediators [6,7]. These compounds act through multiple mechanisms, such as neutralizing reactive oxygen species (ROS), upregulating endogenous antioxidant enzymes including superoxide dismutase (SOD), catalase (CAT), and glutathione peroxidase (GPx), and modulating key inflammatory signaling pathways, notably NF-κB and the NLRP3 inflammasome [8,9]. By attenuating oxidative stress and suppressing the overproduction of pro-inflammatory cytokines such as TNF-α, IL-6, and IL-1β, these bioactive compounds contribute to the protection of neuronal integrity and cognitive function [8].

Brazil has one of the highest global levels of biodiversity and is particularly rich in native fruits containing high concentrations of these bioactive compounds [10]. Fruits such as jaboticaba, açai, camu-camu, tucumã, guarana, cambuci, and macaúba exhibit potent antioxidant and anti-inflammatory activities. Preclinical and clinical studies suggest that their consumption may reduce oxidative stress, modulate inflammatory pathways, and lower the risk of metabolic and neurodegenerative diseases [11,12,13,14]. Moreover, these compounds can influence the gut–brain axis, modulating intestinal microbiota composition and reducing lipopolysaccharide (LPS) translocation, which is known to amplify systemic and cerebral inflammation [8,9].

Likewise, the synergistic antioxidant and anti-inflammatory effects of phytochemicals from native Brazilian fruits suggest a potential “neuroprotective nutrients,” meaning these foods may help restore redox homeostasis, improve mitochondrial function, and reduce microglia and astrocyte-mediated neuroinflammation [8]. “Neuroprotective” encompasses mechanisms that preserve neuronal structure and function by reducing oxidative damage, attenuating microglial or astrocytic activation, and modulating neuroinflammatory cascades. Beyond individual health benefits, incorporating such fruits into sustainable dietary practices may positively impact public health by mitigating the burden of chronic non-communicable diseases both in Brazil and globally [14].

Although several studies have investigated isolated native Brazilian fruits, the available evidence remains fragmented across different experimental models, extraction approaches, and mechanistic outcomes. To date, few reviews have critically integrated the anti-inflammatory and oxidative stress-related mechanisms associated with these fruits within a translational and neuroinflammatory perspective. Therefore, this review aims not only to summarize the phytochemical and biological evidence available but also to critically discuss mechanistic convergence, translational limitations, and current research gaps regarding the use of native Brazilian fruits as potential functional agents against chronic inflammatory disorders.

## 2. Native Brazilian Fruits Investigated in Inflammatory Contexts

Several native Brazilian fruits with anti-inflammatory potential were identified during this review. As for the experimental evidence, 19 native Brazilian fruits were addressed. Jaboticaba (*Plinia cauliflora*) was evaluated in five studies, guarana (*Paullinia cupana*) in four studies, while açai (*Euterpe oleracea* Mart.) and marolo (*Annona crassiflora*) were assessed in two studies. Other species investigated included pinhão manso (*Jatropha curcas*), tomatinho-do-mato (*Solanum diploconos*), jucá (*Libidibia ferrea*), cambuci (*Campomanesia phaea*), goiabão (*Eugenia leitonii*), cereja-do-rio-grande (*Eugenia involucrata*), grumixama (*Eugenia brasiliensis*), pêssego-do-mato (*Eugenia klotzschiana*), genipapo (*Genipa americana*), cutia (*Carpotroche brasiliensis*), macaúba (*Acrocomia aculeata*), blackberry jam (*Randia formosa*), cupuaçu/cupuassu (*Theobroma grandiflorum*), juçara (*Euterpe edulis*) and pitanga (*Eugenia uniflora*), highlighting a broad interest in the bioactive properties of native fruits in experimental research. Table 1 was developed to organize and index the common and scientific names of the native Brazilian fruits included in this review, ensuring taxonomic standardization and facilitating their identification throughout the manuscript.

### 2.1. Jaboticaba (Plinia cauliflora (Mart.) Kausel)

Jaboticaba is one of the most extensively studied native Brazilian fruits in the context of oxidative stress and inflammation. Experimental in vitro and in vivo studies in animals consistently report antioxidant and anti-inflammatory effects associated with phenolic-rich extracts obtained from the peel, whole fruit, leaves, and branches [15,16,17,18,19].

Its biological activity has been primarily attributed to anthocyanins, flavonoids, ellagitannins, and phenolic acids, especially ellagic acid. Overall, these compounds appear to modulate redox-sensitive inflammatory pathways, contributing to reduced oxidative damage and attenuation of inflammatory responses in metabolic and obesity-related conditions [15,18,19].

In addition to the edible fraction, extracts obtained from leaves and branches also demonstrated relevant bioactivity, highlighting the potential of jaboticaba by-products as underexplored sources of functional compounds with possible nutraceutical applications [16].

### 2.2. Guarana (Paullinia cupana var. sorbilis Mart.)

Guarana has been investigated mainly in experimental models exploring the relationship between oxidative stress, inflammation and neurotoxicity. The functional identity of guarana is defined by the synergistic interplay between methylxanthines, such as caffeine, and a dense profile of catechins. Diverging from its conventional use as a stimulant, its biological relevance lies in its neuroprotective axis, particularly in safeguarding neural integrity against chemical and environmental stressors. By attenuating redox-related cellular decay and modulating inflammatory mediators, guarana emerges as a strategic dietary component for preserving cognitive health and preventing neurotoxicity-related tissue damage [20,21,22,23].

### 2.3. Açai (Euterpe oleracea Mart.)

While globally recognized for its antioxidant capacity, the current scientific interest in Açaí has shifted toward its industrial by-products, particularly the seeds. These fractions concentrate high amounts of procyanidins that demonstrate a broad modulation of oxidative and inflammatory responses. In the context of metabolic disorders, açaí acts as a systemic agent capable of attenuating the inflammatory imbalance associated with cardiovascular and renal dysfunction, offering a promising pathway for integrating food waste into high-value nutraceutical applications [24,25].

### 2.4. Marolo (Annona crassiflora Mart.)

The biological potential of Marolo is highlighted by its peel, an underutilized fraction that serves as a concentrated source of flavonoids and phenolic acids. Experimental evidence positions marolo as a facilitator of tissue recovery in preclinical models, where its antioxidant properties translate into a tangible reduction in the inflammatory response. This species represents a significant opportunity for pharmacological prospecting, turning agricultural side-streams into potent agents for mitigating oxidative stress [26,27].

### 2.5. Pinhão-manso (Jatropha curcas L.)

In vivo experimental evidence involving Pinhão-manso investigating leaf-derived extracts rich in phenolic compounds and flavonoids [12] demonstrated anti-inflammatory activity in neuroinflammatory models, suggesting possible modulation of oxidative and inflammatory pathways associated with neural damage. Although still preliminary, these findings indicate the potential of J. curcas as a source of bioactive compounds with possible neuroprotective applications.

### 2.6. Tomatinho-do-mato (Solanum diploconos (Mart.) Bohs)

Tomatinho-do-mato has demonstrated a high content of bioactive compounds, particularly phenolic compounds, as well as vitamin C and carotenoids, indicating a strong antioxidant profile. The available evidence is restricted to in vitro antioxidant assays using whole-fruit extracts, which demonstrated relevant free radical scavenging activity. However, anti-inflammatory effects have not yet been directly investigated in biological models [28].

### 2.7. Jucá/Pau-ferro (Libidibia ferrea (Mart. ex Tul.) L.P.Queiroz)

The pharmacological relevance of *L. ferrea* is primarily centered on its specialized protein fractions, notably purified trypsin inhibitors found in its seeds. Beyond traditional uses, these compounds demonstrate a sophisticated ability to attenuate both inflammatory and nociceptive responses. The unique action of jucá in modulating pain and acute inflammation markers highlights its potential as a source of bioactive peptides that bridge the gap between traditional medicine and modern biopharmaceutical development [29].

### 2.8. Cambuci (Campomanesia phaea (O. Berg) Landrum)

Cambuci serves as a strategic nutritional model for addressing the complexities of obesity-induced inflammation. Its polyphenol-rich pulp acts as a metabolic stabilizer, capable of correcting the oxidative imbalance that characterizes chronic low-grade inflammatory states. By improving metabolic parameters in high-fat diet models, cambuci is positioned as a functional fruit with the potential to mitigate the systemic triggers of metabolic syndrome through dietary intervention [30].

### 2.9. Goiabão (Eugenia leitonii D.Legrand)

In an in vivo animal model comparative study of four *Eugenia* species (*Eugenia leitonii*, *E. involucrata*, *E. brasiliensis*, and *E. myrcianthes*), Infante et al. [31] identified high phenolic content and antioxidant activity for Goiabão, especially in seed-derived extracts rich in gallic acid and epicatechin. Experimental findings also indicated reduced neutrophil migration, pointing to possible anti-inflammatory activity. Although evidence remains limited, the available data highlight goiabão as a promising native source of bioactive compounds.

### 2.10. Cereja do Rio Grande (Eugenia involucrata DC.)

Similarly, Infante et al. [31] reported antioxidant activity in leaves of Cereja-do-rio-grande (*Eugenia involucrata*), particularly in extracts rich in epicatechin and gallic acid. Leaf-derived extracts also reduced neutrophil migration in experimental models, whereas pulp and seed fractions showed lower activity. Although evidence remains limited, these findings suggest potential antioxidant and anti-inflammatory properties associated mainly with the leaves.

### 2.11. Grumixama (Eugenia brasiliensis Lam.)

Unlike other *Eugenia* species, Grumixama stands out because its pulp maintains an antioxidant potency comparable to its leaves, a rare trait among native fruits. This biological activity is largely attributed to a high concentration of gallic acid and epicatechin. On top of to its radical scavenging capacity, Grumixama extracts have demonstrated significant anti-inflammatory effects in vivo, particularly in modulating neutrophil migration. The efficacy of these extracts, often comparable to reference anti-inflammatory drugs like dexamethasone, suggests a synergistic action between their phenolic compounds that targets both oxidative damage and cellular recruitment in inflammatory processes [31].

### 2.12. Pêssego-do-mato (Eugenia myrcianthes Nied)

According to the authors [31], the therapeutic potential of Pêssego-do-mato is predominantly driven by its high epicatechin and gallic acid content, which translates into a potent dual action: direct neutralization of reactive species—notably superoxide radicals—and a systemic attenuation of the inflammatory cascade. A critical observation regarding this species is the tissue-dependent nature of its bioactivity; while the pulp and leaves demonstrate anti-inflammatory efficacy comparable to reference corticosteroids, the seeds appear to lack such properties. This distinction is vital for future pharmacological prospecting, as it suggests that the protective effects are concentrated in the edible portions and their associated metabolic fractions rather than the entire botanical matrix.

### 2.13. Genipapo (Genipa americana L.)

Diverging from most phenolic-rich fruits, the biological relevance of genipapo is centered on its iridoid content, specifically genipin. Its value is particularly noteworthy in the field of neuroprotection, where it demonstrates a robust capacity to preserve functional reactivity against cholinergic decline. This species fills a critical gap by offering a multifaceted approach that combines anticholinesterase activity with protein cross-linking properties, positioning genipapo as a unique nutraceutical candidate for preserving cognitive health in neurodegenerative diseases [32].

### 2.14. Cutia (Carpotroche brasiliensis (Raddi) A. Gray)

The seed oil of the cutia fruit occupies a singular chemical niche due to the presence of cyclopentenyl fatty acids, historically associated with the treatment of complex inflammatory conditions. Its mechanistic relevance focuses on the modulation of peripheral pain and inflammation pathways, acting effectively without the gastric adverse effects common to synthetic non-steroidal anti-inflammatory drugs. This pharmacological specificity directs the use of cutia toward the management of acute tissue responses and peripheral pain, scientifically validating its traditional medicinal use [33].

### 2.15. Macaúba (Acrocomia aculeata (Jacq.) Lodd. ex Mart.)

Macaúba acts as a strategic metabolic modulator through the integration of monounsaturated fatty acids with a lipophilic matrix of carotenoids and tocopherols. More than just a lipid source, its oil operates at the intersection of oxidative stress and adipogenesis, demonstrating a unique capacity to reprogram the inflammatory response within adipose tissue. By mitigating adipocyte hypertrophy and macrophage infiltration, macaúba emerges as an essential nutritional tool for controlling the low-grade inflammation associated with metabolic syndrome [34].

### 2.16. Blackberry Jam (Randia formosa (Jacq.) K. Schum)

The biological importance of Blackberry Jam Fruit lies in its selective and dual action within the central nervous system. Its phytochemical profile facilitates a multifaceted defense that combines the mitigation of protein oxidative damage with a highly selective inhibitory affinity for butyrylcholinesterase. This selectivity is a crucial pharmacological trait for addressing advanced stages of neurodegeneration, elevating the fruit from a simple dietary antioxidant to a targeted agent for maintaining neurological integrity [35].

### 2.17. Juçara (Euterpe edulis Mart.)

Juçara transcends the role of a radical scavenger by demonstrating the ability to orchestrate a shift in the cellular inflammatory microenvironment. Through the rebalancing of the cytokine network, this fruit facilitates the transition of macrophages from a pro-inflammatory state toward a resolutive profile. This immunomodulatory capacity positions juçara as a pivotal functional ingredient for managing chronic inflammation, offering a proactive approach to stabilizing cellular homeostasis [36].

### 2.18. Cupuaçu/Cupuassu (Theobroma grandiflorum (Willd. ex Spreng.) K. Schum.)

Cupuaçu serves as a model of how fermentation-mediated biotransformation can amplify the biological efficacy of native matrices. Microbial synergy unlocks more bioavailable alkaloid and flavonoid profiles, translating into a robust systemic defense. In the context of acute inflammatory challenges, fermented cupuaçu acts as a sophisticated immunomodulator, stabilizing physiological parameters and regulating leukocyte recruitment, thus standing out for its enhanced protective capacity [37].

### 2.19. Pitanga (Eugenia uniflora L.)

Pitanga offers a specialized perspective on barrier immunity and mucosal protection. Its relevance lies in the capacity to provide immediate and localized protection, acting as an active barrier against bacterial challenges, particularly in the oral cavity. By stabilizing the gingival environment and preventing the onset of localized inflammatory processes, pitanga integrates nutrition into oral health maintenance, filling an important gap in the prevention of chronic tissue damage through accessible dietary interventions [38].

Table 2 was constructed to synthesize individual experimental studies investigating the anti-inflammatory and redox-related effects of Brazilian native fruits. Due to the heterogeneity of experimental designs, extraction methods, and biological models, the reported doses and units follow those described in the original studies.

## 3. Phytochemical Composition

Analysis of the bioactive composition reported in the included studies (Table 2) revealed that phenolic compounds were by far the most frequently identified class, appearing in 20 out of the 25 experimental studies. Within this class, flavonoids and phenolic acids were the dominant phenolic groups, consistently reported across different fruits, plant parts, and experimental models.

Flavonoids were the most prevalent phenolic subclass, being identified in 15 studies. Among flavonoid subgroups, catechin and quercetin were the most frequently reported. Catechin was identified in 7 studies, notably in cambuci (*Campomanesia phaea*), marolo (*Annona crassiflora*), and multiple studies involving guarana (*Paullinia cupana*). Quercetin was also reported in 7 studies, including cambuci, jaboticaba (*Plinia* spp.), and marolo.

Other flavonoids occurred less frequently but remained recurrent across species. Epicatechin appeared in 5 studies, predominantly in *Eugenia* species and guarana-based extracts. Rutin appeared in 3 studies, while myricetin was identified in 2 studies, mainly in jaboticaba-derived extracts. Anthocyanins, although restricted to fewer species, were consistently reported in 4 studies, all involving jaboticaba, particularly in peel and whole-fruit preparations.

Phenolic acids constituted the second most prevalent phenolic subgroup, reported in 14 studies. Among these, gallic acid was the most frequently cited individual compound, appearing in 6 studies, notably in cambuci, marolo, jaboticaba, and *Eugenia* species. Ellagic acid was identified in 5 studies, almost exclusively associated with jaboticaba extracts. Phenolic-acid derivatives and hydrolyzable tannins, including gallotannins and ellagitannins, were described in 5 studies, again predominantly in jaboticaba-based investigations.

Beyond flavonoids and phenolic acids, tannins were explicitly reported in 4 studies, particularly in jaboticaba and guarana extracts, reinforcing their contribution to antioxidant and anti-inflammatory effects. Polymeric proanthocyanidins were associated with açai (*Euterpe oleracea*), focused on seed extracts in 2 studies, and cambuci, on pulp polyphenol extracts (*Campomanesia phaea*), appearing in 3 studies.

Although less frequent, several non-phenolic bioactive compounds were also identified. Carotenoids were reported in 2 studies, primarily in tomatinho-do-mato (*Solanum diploconos*) and macaúba (*Acrocomia aculeata*), while tocopherols appeared in 2 studies, both in lipid-rich fruit matrices. Fatty acids, including oleic acid, were identified in 3 studies, involving macaúba (*Acrocomia aculeata*), cutia fruit (*Carpotroche brasiliensis*), and juçara (*Euterpe edulis*).

Some compounds demonstrated marked specificity. Saponins were reported in 1 study, exclusively in pinhão-manso (*Jatropha curcas*). Genipin appeared in 1 study, restricted to genipapo (*Genipa americana*). Coumarins and lactones were identified in 1 study involving Blackberry Jam Fruit (*Randia formosa*). Finally, protease inhibitors, represented by the compound LfTI, were uniquely reported in 1 study with jucá (*Libidibia ferrea*).

Taken together, these findings demonstrate that flavonoids and phenolic acids are the primary and most consistently reported bioactive groups in native Brazilian fruits, with gallic acid, quercetin, catechin, and epicatechin emerging as the most recurrent individual compounds. The biological relevance of these species is rooted in this potent phenolic assembly, which provides more than just a foundational antioxidant defense. These compounds act as the central drivers of metabolic and neurological protection, operating through a multifaceted network of redox stabilization and enzyme modulation.

While the phenolic core provides the fundamental therapeutic strength, the presence of chemically diverse non-phenolic constituents—such as iridoids, specialized fatty acids, and protease inhibitors—further amplifies this potential. These rare metabolites act in concert with the phenolic matrix, engaging with specific pathways like cholinergic signaling or adipocyte reprogramming. Consequently, the efficacy of native Brazilian fruits is defined by this phenolic-led synergy. It positions these fruits as sophisticated functional matrices where a high concentration of established polyphenols works alongside unique secondary metabolites, reinforcing their critical role as a strategic resource for managing chronic low-grade inflammation and metabolic health.

Table 3 presents data obtained from a search of the scientific literature regarding the quantification of bioactive compounds and the antioxidant activity of the fruits included in this review. Studies that performed quantitative analyses of bioactive composition as well as evaluations of antioxidant capacity using different analytical methods were selected, allowing comparisons among matrices, processing forms, and methodological approaches. This compilation aims to synthesize the available evidence and provide an overview of the reported values, contributing to the understanding of the functional potential of these fruits.

Overall, the analyzed studies demonstrate wide variability in the contents of bioactive compounds and antioxidant activity values, which can be attributed to factors such as species, the part of the fruit analyzed, processing methods, extraction procedures, and analytical techniques. Nevertheless, the compiled data reinforce the high antioxidant potential of the studied fruits, highlighting their nutritional and functional relevance. The systematization of this information enables the identification of gaps in the literature and supports future investigations focused on methodological standardization and the application of these compounds in health promotion.

The metabolization and bioavailability of bioactive compounds present in native Brazilian fruits are determining factors for their biological efficacy. Although these fruits contain a high density of phenolic compounds, carotenoids and terpenoids with demonstrated antioxidant and anti-inflammatory potential, their effects depend fundamentally on release, absorption, metabolic transformation, and systemic distribution [12,15,26,49]. Soluble phenolics such as flavonoids and simple phenolic acids are partially absorbed in the small intestine, but their bioavailability is limited by hepatic conjugation processes—sulfation, methylation, and glucuronidation—as well as by extensive microbial metabolism in the colon [17,50,51,52]. Phenolic compounds from fruits such as jaboticaba, cambuci and marolo undergo intense intestinal and hepatic biotransformation before reaching systemic circulation, directly influencing their anti-inflammatory and antioxidant actions [15,30].

A central aspect of this metabolic process is the participation of the gut microbiota. Complex phenolics, particularly tannins and ellagitannins, are converted by intestinal microbes into smaller metabolites—such as phenolic acids and urolithins—that display significantly enhanced bioactivity and bioavailability. These microbial-derived metabolites have been associated with the modulation of inflammatory pathways, improvement of intestinal barrier integrity, and attenuation of systemic and neuroimmune responses, although their formation varies considerably between individuals due to differences in diet, age, microbiota composition and health status [51,52,53]. Growing evidence indicates that, in many cases, the physiological effects of polyphenol-rich fruits are mediated more by these microbial metabolites than by the parent compounds themselves, including modulation of NF-κB and NLRP3 activity and mitigation of oxidative stress [54]. This reinforces the concept that polyphenols exert prebiotic-like actions, shaping microbial communities and, reciprocally, being transformed into highly active metabolites.

Colonic fermentation is also essential for the release of insoluble-bound phenolics, further contributing to interindividual variability in metabolite formation [12,52,53]. Carotenoids present in native species—such as β-carotene, lutein, zeaxanthin and lycopene—exhibit bioavailability dependent on lipid intake, micellar incorporation and intestinal membrane integrity. After absorption, they are transported in chylomicrons and distributed primarily to the liver, skin and retina, with processing techniques enhancing their bioaccessibility [17]. Genipin, a key iridoid from genipapo, also demonstrates promising intestinal permeability and metabolic stability, though clinical pharmacokinetic data remain scarce [32].

Despite the promising bioactivity demonstrated by native Brazilian fruits in preclinical models, a significant translational gap remains regarding their pharmacokinetics and metabolic fate. Current evidence is predominantly derived from in vitro or acute in vivo studies, which often bypass the complex interactions within the human gastrointestinal tract. Most notably, there is an almost complete absence of data concerning the microbial metabolism, enteric absorption, plasma kinetics, or tissue distribution of these unique phytochemical matrices. This lack of information represents a critical bottleneck; without understanding how the colonic microbiota transforms parent compounds into bioactive metabolites, it is impossible to accurately determine systemic exposure or identify the true biological effector molecules.

This ‘metabolic black box’ severely limits the transposition of experimental findings into clinical dietary recommendations. While a fruit may exhibit potent antioxidant or neuroprotective effects in a controlled environment, its real-world efficacy is governed by bioavailability, which is notoriously low for many polyphenols, and high inter-individual variability in gut microbiota composition. Such variability suggests that the health benefits of native fruits may not be universal but rather dependent on specific host metabotypes. Consequently, moving beyond descriptive science requires future research to incorporate integrated metagenomic, metabolomic, and pharmacokinetic approaches. Establishing whether these therapeutic effects arise from parent compounds, microbial metabolites, or their synergistic interplay is essential for developing evidence-based nutraceutical strategies that reflect the metabolic reality of the human holobiont.

## 4. Toxicity and Safety

Overall, the evidence derived from the analyzed experimental studies suggests that Brazilian native fruits present a favorable safety profile, particularly within the tested doses and experimental conditions. Most studies reported the absence of cytotoxicity or low toxicity, reinforcing their potential for use in nutritional and therapeutic applications.

For jaboticaba (*Plinia cauliflora*), no toxicity was observed at high doses (up to 2000 mg/kg) in vivo [15], while in vitro assays demonstrated no cytotoxicity at most concentrations, with only slight reductions at the highest tested dose (125 μg/mL) [16]. Similarly, studies with açaí (*Euterpe oleracea*) [24,25], cambuci (*Campomanesia phaea*) [30], macaúba (*Acrocomia aculeata*) [34], and mixed *Eugenia* species [31] did not report toxicity, although in some cases, toxicity endpoints were not explicitly evaluated.

For guaraná (*Paullinia cupana*), the data indicate a dose-dependent safety profile. In vitro studies showed no relevant cytotoxicity at lower concentrations, with a slight reduction in cell viability at ≥10 mg/mL [20], and no cytotoxicity across a range of 10–300 μg/mL [21]. In vivo, no toxicity was observed at doses up to 10 mg/mL, but reduced viability was reported at 20 mg/mL, suggesting a hormetic response at higher concentrations [22]. A possible explanation for the observed cytotoxic effects at higher concentrations may be related to the presence of caffeine and flavonoids, which, depending on the dose, can exert pro-oxidant or cytotoxic effects.

Marolo (*Annona crassiflora*) [26,27], pinhão manso (*Jatropha curcas*) [12] and jucá (*Libidibia ferrea*) [29] consistently demonstrated the absence of cytotoxicity in vitro, even at relatively high concentrations, indicating good cellular tolerance.

For genipapo (*Genipa americana*), no toxicity was reported based on literature data [32], while pitanga (*Eugenia uniflora*) showed no cytotoxicity under both pre-treatment and co-treatment conditions in human gingival fibroblasts [38], supporting its safety in short-term exposure models.

An exception among the analyzed fruits is cutia fruit (*Carpotroche brasiliensis*), which demonstrated a dose-dependent toxic effect in vivo. While no mortality was observed at doses up to 300 mg/kg (with only mild lethargy), a 30% mortality rate occurred at 500 mg/kg, indicating a relatively narrow safety margin at higher doses [33].

For juçara (*Euterpe edulis*), most fractions were non-cytotoxic (1–100 μg/mL), although specific fractions (dichloromethane and butanol) exhibited cytotoxicity at 100 μg/mL, highlighting the importance of the extraction method and compound polarity in safety assessment [36].

Importantly, several studies did not report toxicity outcomes, which represents a methodological limitation and highlights the need for more comprehensive toxicological evaluations, especially chronic exposure studies and standardized dose–response assessments.

In summary, the current body of evidence indicates that Brazilian native fruits are generally safe within physiologically relevant doses, with occasional dose-dependent or fraction-specific cytotoxic effects. These findings support their potential application in functional foods and nutraceuticals while also emphasizing the importance of dose optimization and detailed toxicological characterization in future studies.

## 5. Biological Effects

**Figure 1:**
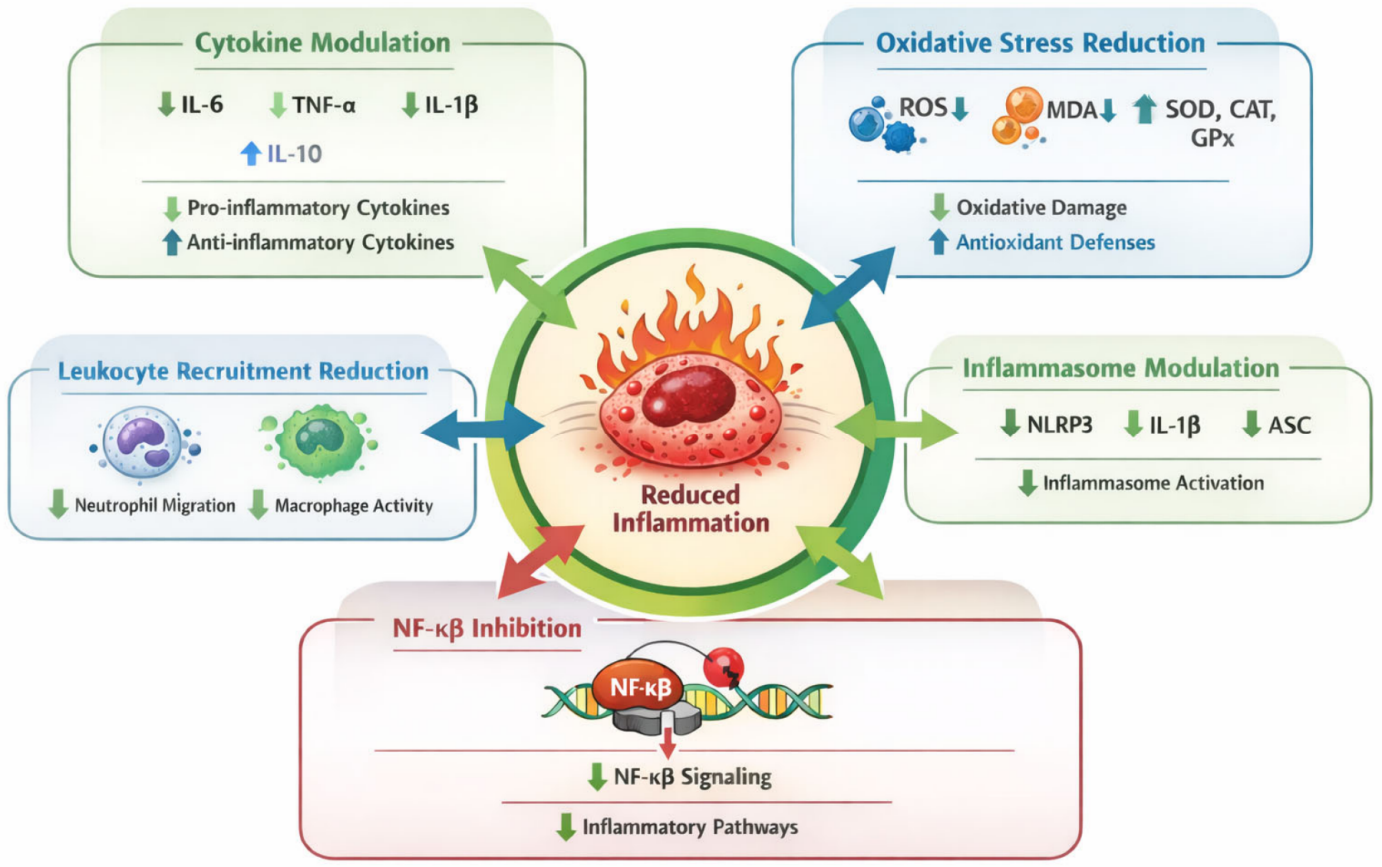
Predominant anti-inflammatory mechanisms associated with native Brazilian fruits.

Across the 25 included studies (Table 2), the anti-inflammatory effects of native Brazilian fruits converged on a limited number of recurrent mechanisms (Figure 1), primarily involving cytokine modulation, oxidative stress control, leukocyte recruitment, and regulation of key inflammatory signaling pathways. In addition, the quantification of antioxidant activity values of these fruits is presented in Table 3, providing a complementary overview of their bioactive potential.

### 5.1. Mechanisms of Action in Modulating Inflammation

Suppression of pro-inflammatory cytokines was the most frequently reported mechanism, observed in 13 studies. Reductions in IL-6 were described in studies with jaboticaba [16,17,19], guarana [22,23], cambuci [30], marolo [26,27], macaúba [34] and açai [24,25]. Decreases in TNF-α were reported in 7 studies, including cambuci [30], marolo [26,27], macaúba [34], açai [24,25], and guarana [22,23]. Reductions in IL-1β were observed in 5 studies, particularly in jaboticaba-based interventions [17,18] and guarana [22,23]. In contrast, upregulation of anti-inflammatory cytokines, notably IL-10, was reported in a study involving guarana supplementation [22].

Modulation of leukocyte recruitment and inflammatory cell activity was described in 9 studies. Reductions in neutrophil migration or influx were reported in studies involving *Eugenia* species [31], marolo [27], jaboticaba [15], and cutia fruit [33]. Decreased macrophage activation or activity was documented in marolo-derived phenolic fractions [27] and jaboticaba extracts [16].

Attenuation of oxidative stress represented another highly prevalent mechanism, reported in 14 studies. Reductions in ROS or lipid peroxidation markers—such as MDA—were observed in blackberry jam [35], macaúba [34], jaboticaba [17,18], açai [24,25], guarana [20,23], tomatinho do mato [28] and pitanga [38].

Enhancement of endogenous antioxidant defenses was documented in 10 studies, with increases in SOD reported in macaúba [34], açai [24,25], guarana [20,21,23], blackberry jam [35], and pitanga [38]. Increases in CAT were observed in 4 studies, particularly in guarana [21], açai [24,25], and blackberry jam [35], while GPx upregulation was reported in 1 study involving açai [24,25].

Direct inhibition of NF-κB signaling was explicitly reported in 4 studies, including cambuci [30], jaboticaba [16,17,19], macaúba [34], and pinhão-manso [12]. In these studies, NF-κB suppression was accompanied by reductions in downstream inflammatory mediators such as cytokines and nitric oxide

Inflammasome-related pathways were less frequently reported but showed high mechanistic specificity. Downregulation of NLRP3 inflammasome components, including NLRP3, ASC, and IL-1β, was documented in 1 study involving jaboticaba polyphenol extracts [17], indicating targeted inhibition of inflammasome-dependent inflammation.

Several studies also demonstrated neuroinflammatory modulation, reported in 4 studies. Reductions in microglial and astrocytic activation markers (Iba-1 and GFAP) and inhibition of NO production were described for pinhão-manso leaf extracts [12]. Neuroprotective and anti-inflammatory effects were also observed in studies involving guarana [22,23], jaboticaba [17], and blackberry jam, which additionally showed anticholinesterase activity [35].

At the systemic level, improvements in inflammation-associated metabolic outcomes, such as reductions in edema, vascular permeability, insulin resistance, and adipogenesis, were reported in 7 studies, particularly those involving macaúba oil [34], jaboticaba [15,19], açai [24,25], cambuci [30], and marolo [26,27].

Overall, the quantitative synthesis of inflammatory mechanisms indicates that native Brazilian fruits exert their anti-inflammatory effects predominantly through suppression of pro-inflammatory cytokines [19,26,30], attenuation of oxidative stress [18,34,35], and reduction in leukocyte recruitment [27,31]. Less frequent but mechanistically relevant pathways include NF-κB inhibition [12,30] and inflammasome modulation [17], together supporting a broad immunomodulatory profile extending from peripheral immune regulation to neuroinflammatory control.

### 5.2. Antioxidants in the Modulation of Inflammation

Inflammation and oxidative stress are closely interconnected pathophysiological processes that participate in a self-perpetuating cycle in the development of several chronic and neurodegenerative diseases. The excess production of ROS and nitrogen species (RNS), combined with the overproduction of pro-inflammatory cytokines, results in oxidative damage to lipids, proteins, and DNA, besides activating molecular pathways that sustain inflammation, such as nuclear factor kappa B (NF-κB) and the NLRP3 inflammasome. In the central nervous system, these mechanisms contribute to chronic microglial activation, establishing a state of neuroinflammation that, when persistent, promotes synaptic dysfunction and neuronal degeneration [8,9].

Evidence from preclinical studies in this review suggests that bioactive compounds found in native Brazilian fruits, particularly polyphenols, may contribute to the simultaneous attenuation of these processes. These compounds act as primary antioxidants by neutralizing free radicals and as secondary modulators by inhibiting pro-oxidant enzymes and inducing the expression of endogenous defense enzymes, including SOD, CAT, and GPx. In addition, they modulate the NF-κB signaling pathway, reducing the transcription of inflammatory mediators such as TNF-α, IL-6, and IL-1β.

The antioxidant effects described in the included studies are consistent with the broader literature on polyphenols, which indicates that these compounds act not only through direct free radical scavenging but also by downregulating oxidative pathways involved in inflammation [6,30,52]. This combination of mechanisms helps explain the reductions in MDA and the increases in enzymatic antioxidant defenses such as SOD, CAT, and GPx observed across several of the fruits evaluated. Moreover, specific phenolics, including gallic and ellagic acids and various anthocyanins, exhibit strong reducing capacity due to their conjugated aromatic systems and multiple hydroxyl substitutions. These structural attributes support the robust antioxidant activity demonstrated in assays such as DPPH, ABTS, and FRAP [6,50].

The study by Nagpal et al. [8] reinforces that dietary antioxidants have the potential not only to prevent but also to modulate the progression of neurodegenerative disorders through mitochondrial and immunoneural mechanisms. These authors emphasize that diets rich in phenolic compounds promote neuroprotection by restoring redox homeostasis, improving mitochondrial function, and reducing microglia and astrocyte-mediated neuroinflammation. Similarly, Silla et al. [9] demonstrated that antioxidant compounds derived from citrus waste, such as hesperidin, naringenin, and nobiletin, suppress the expression of NF-κB, COX-2, and iNOS while promoting the anti-inflammatory polarization of microglia and astrocytes, indicating a mechanism comparable to that observed in flavonoid-rich Brazilian fruits.

These findings are consistent with those of the present review, which reveal similar effects of native fruits in modulating inflammatory and oxidative pathways. For example, polyphenols from jaboticaba and cambuci inhibit the NLRP3 inflammasome and NF-κB activation; carotenoids and macaúba enhance SOD and CAT activity; and flavonoids from blackberry jam fruit reduce pro-inflammatory cytokines while improving metabolic and cognitive parameters. Thus, the synergistic antioxidant and anti-inflammatory actions of these compounds appear to function as a protective axis against neuroinflammation [8].

Beyond their direct action on molecular pathways, there is evidence that these compounds influence the gut–brain axis, modulating intestinal microbiota and consequently reducing LPS translocation, a factor that amplifies systemic and cerebral inflammation [9]. This mechanism represents an emerging pathway in the prevention of inflammation-associated neurodegeneration.

### 5.3. Impact on Neuroinflammation

Neuroinflammation is an inflammatory response within the central nervous system (CNS), mediated primarily by activated microglia and astrocytes. In its acute form, it is essential for neural protection; however, when chronically activated, it becomes a key factor in the development of psychiatric and neurodegenerative disorders [3]. Increasing evidence indicates that systemic inflammation often acts as a trigger for this process. Circulating pro-inflammatory cytokines, such as interleukin-6 (IL-6), tumor necrosis factor-alpha (TNF-α), and interleukin-1β (IL-1β), can cross the blood–brain barrier, particularly when its integrity is compromised—activating glial cells and sustaining a persistent inflammatory cycle in the CNS [3,55,56].

Systemic inflammation is closely associated with chronic diseases such as cardiovascular disease, obesity, and asthma, which share a common mechanism of inflammatory dysregulation. One of the main drivers of this condition is the Western dietary pattern, considered obesogenic due to its promotion of pro-inflammatory biochemical mediators. Excessive caloric intake and high consumption of ultra-processed foods favor lipid accumulation and adipocyte stress, shifting the balance of adipokines and cytokines toward a pro-inflammatory profile. This inflammatory state can be monitored through biomarkers, such as C-reactive protein (CRP), IL-6, TNF-α, and reduced levels of anti-inflammatory mediators, including adiponectin [3,55,56,57].

The relationship between inflammation and mental health outcomes is well established. Meta-analyses demonstrate that individuals with depression frequently present elevated systemic inflammatory markers, such as CRP, IL-6, and TNF-α, even in the absence of organic comorbidities [58]. Likewise, clinical studies administering cytokines, such as interferon-α, for the treatment of hepatitis C or cancer show that approximately one-third of treated patients develop depressive symptoms, highlighting a causal role of inflammation in depression pathophysiology [59]. Pro-inflammatory cytokines interfere with dopaminergic and serotonergic neurotransmission and impair hippocampal neurogenesis, contributing to symptoms such as anhedonia, fatigue, and psychomotor slowing [60,61]. Similar inflammatory and neuroinflammatory signatures are also observed in schizophrenia and Alzheimer’s disease, suggesting that chronic inflammation contributes to neurodegeneration and synaptic disorganization [4,61].

Dietary strategies aimed at reducing systemic inflammation emerge as promising modulators of neuroinflammatory processes. Native Brazilian fruits, rich in polyphenols and other bioactive compounds, have shown significant potential to regulate inflammatory signaling pathways involved in neuroinflammation, particularly NF-κB activation, NLRP3 inflammasome assembly, oxidative stress, and glial reactivity [6,10,13,49].

Guarana predominantly exerts neuroprotective effects via attenuation of oxidative stress and suppression of pro-inflammatory cytokines, with consistent reductions in TNF-α, IL-1β, and IL-6, alongside increased activity of antioxidant enzymes such as SOD and CAT [22]. These effects indicate a primary action on redox-sensitive inflammatory pathways, indirectly limiting microglial activation and protecting neuronal integrity. Evidence of NF-κB modulation further supports guarana’s role in controlling upstream inflammatory transcriptional processes [20,21,62].

Pinhão-manso, in turn, combines phenolic compounds with terpenoids, steroids, and saponins and exhibits a distinct neuroinflammatory profile characterized by direct modulation of glial cell activation. Leaf extracts of *Jatropha curcas* significantly reduced microglial (Iba-1) and astrocytic (GFAP) activation markers, as well as NO production, in LPS-stimulated glial and macrophage models [12]. This pattern indicates a cell-specific anti-neuroinflammatory action, targeting the effector cells responsible for sustaining chronic neuroinflammation.

Additionally, neuroprotective effects associated with native Brazilian fruits may extend beyond classical anti-inflammatory pathways by involving modulation of oxidative stress, cholinergic signaling, and neuronal defense systems. Experimental evidence from Blackberry Jam fruit [35] demonstrated that its pulp extract significantly reduced lipid peroxidation and protein carbonylation in murine brain tissue exposed to H_2_O_2_-induced oxidative stress, while simultaneously increasing the activity of endogenous antioxidant enzymes such as SOD and CAT. The extract also inhibited acetylcholinesterase (AChE) and butyrylcholinesterase (BChE), mechanisms commonly associated with preservation of cholinergic neurotransmission and attenuation of cognitive decline in neurodegenerative disorders. These effects were attributed to the presence of bioactive compounds, including flavonoids, coumarins, sphingosines, and epicatechin derivatives, which are known to modulate redox-sensitive pathways and neuronal survival. Importantly, the reduction in oxidative damage biomarkers suggests potential indirect suppression of neuroinflammatory cascades, since oxidative stress and chronic glial activation are closely interconnected processes in the pathophysiology of depression, Alzheimer’s disease, and other neuropsychiatric disorders.

Genipin, an iridoid compound found in *Genipa americana* L., has also demonstrated promising neuroprotective potential through antioxidant, anti-inflammatory, and anticholinesterase mechanisms. Experimental studies [32] suggest that genipin and related iridoids may help preserve neuronal function by reducing oxidative stress, limiting neuroinflammatory damage, and increasing acetylcholine availability through inhibition of AChE and BChE. In addition, these compounds have been associated with reduced amyloid aggregation, inhibition of tau hyperphosphorylation, and protection against synaptic dysfunction, mechanisms closely related to the pathophysiology of Alzheimer’s disease and cognitive decline. The presence of other bioactive compounds co-extracted from *Genipa americana* may further contribute to these effects through synergistic antioxidant and neuroprotective actions.

Although some bioactive compounds present in native Brazilian fruits have demonstrated the ability to inhibit AChE and BChE [32,35], this mechanism should be interpreted with caution in the context of neuroprotection. Cholinesterase inhibitors are widely used as palliative treatments in neurodegenerative diseases, particularly Alzheimer’s disease, due to their capacity to increase acetylcholine availability and improve cognitive symptoms [32]. However, these effects are primarily symptomatic rather than disease-modifying. Furthermore, excessive inhibition of AChE may lead to sustained cholinergic stimulation and neuronal hyperexcitation, suggesting that this mechanism does not necessarily confer direct neuroprotective effects. Therefore, while anticholinesterase activity may contribute to functional improvements, its role in modulating neuroinflammation and preventing neurodegeneration remains uncertain and likely depends on dose, biological context, and interaction with other antioxidant and anti-inflammatory pathways.

**Figure 2:**
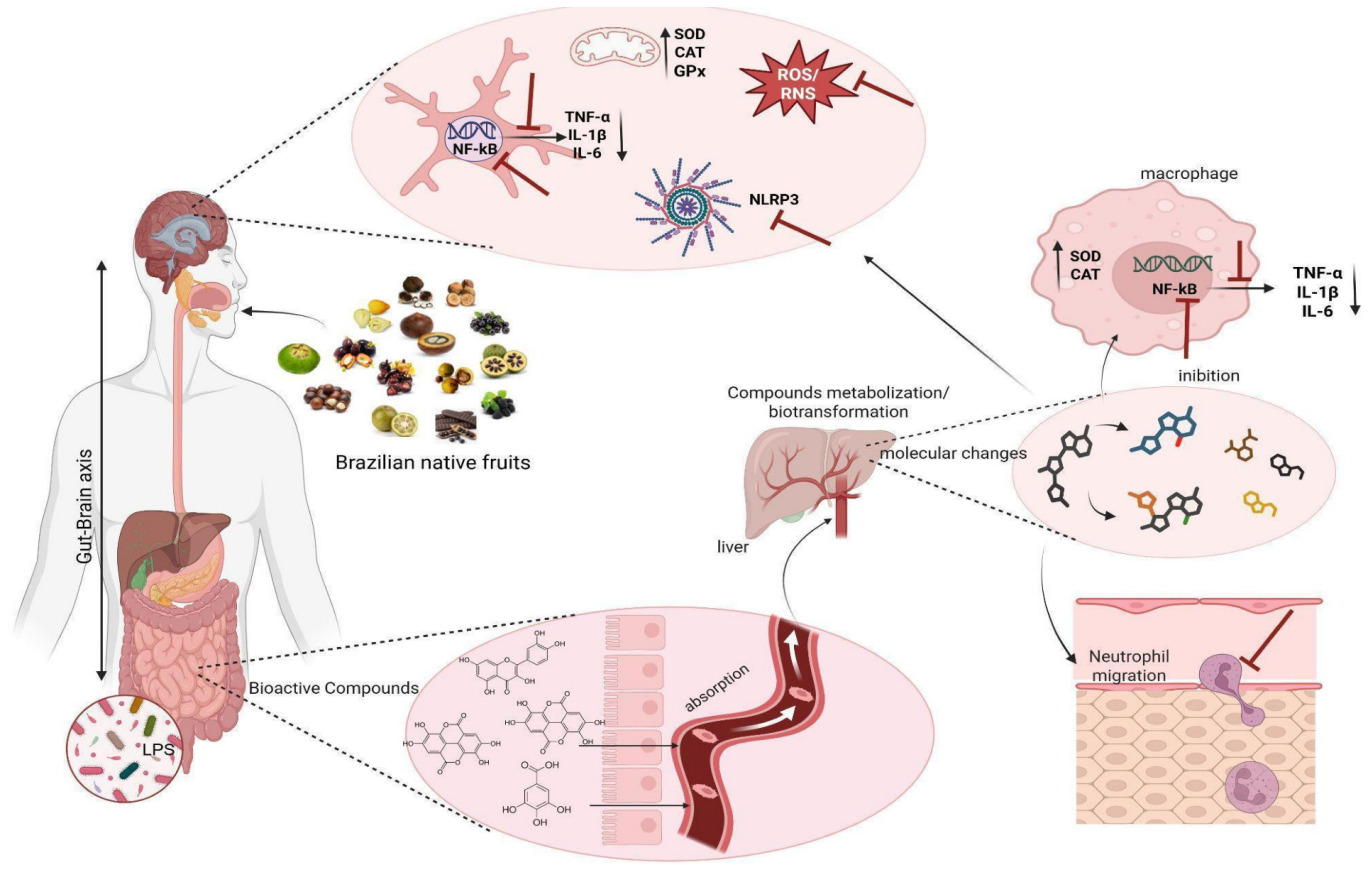
Effects of bioactive compounds from Brazilian native fruits on inflammation and the gut–brain axis.

Taken together, the available evidence suggests that native Brazilian fruits may modulate neuroinflammation through multiple and complementary mechanisms involving reduction in oxidative stress, suppression of pro-inflammatory cytokines, regulation of NF-κB and NLRP3 signaling pathways, attenuation of glial activation, and preservation of neuronal homeostasis. In addition to direct effects within the CNS, these fruits may also influence systemic inflammatory responses through gut-derived and metabolic pathways, reinforcing the relevance of the gut–brain axis in neuropsychiatric and neurodegenerative disorders. Bioactive compounds such as polyphenols, iridoids, flavonoids, and carotenoid-related metabolites appear to act synergistically to reduce ROS/RNS production, improve endogenous antioxidant defenses, and limit chronic inflammatory signaling. Figure 2 summarizes these proposed mechanisms, illustrating how bioactive compounds from native Brazilian fruits may be absorbed and metabolized, subsequently modulating peripheral immune responses, oxidative stress pathways, cytokine production, macrophage activation, neutrophil migration, and neuroinflammatory signaling within the brain. Collectively, these findings support the potential of native Brazilian fruits as promising dietary strategies for the prevention and modulation of inflammation-related mental and neurodegenerative disorders, although further clinical and translational studies remain necessary to confirm these effects in humans.

### 5.4. Impact on Airway Inflammation

Modulation of leukocyte recruitment and inflammatory cell activity, particularly involving neutrophils and macrophages, was consistently reported across several studies included in this review. These mechanisms are not only central to systemic inflammation but also play a critical role in the pathophysiology of chronic airway inflammatory diseases, such as chronic obstructive pulmonary disease (COPD) and asthma. Neutrophil infiltration, excessive production of reactive oxygen species (ROS), and sustained release of pro-inflammatory cytokines are hallmark features of these conditions and contribute to progressive tissue damage and impaired pulmonary function [63,64].

Environmental factors, particularly air pollution, are well-established triggers of airway inflammation through oxidative stress and activation of innate immune responses. Exposure to particulate matter (PM2.5) and other pollutants promotes leukocyte recruitment, epithelial injury, and activation of inflammatory signaling pathways, including NF-κB and MAPK, thereby exacerbating respiratory diseases [65]. In this context, dietary antioxidants have emerged as potential modulators of pollution-induced oxidative stress and inflammation.

Although the primary focus of the studies included in this review was systemic and metabolic inflammation, the observed ability of native Brazilian fruits to reduce leukocyte migration, attenuate oxidative stress, and modulate pro-inflammatory cytokines suggests a potential role in mitigating airway inflammatory responses. Bioactive compounds such as flavonoids, phenolic acids, and carotenoids, widely identified in these fruits, have been shown to exert protective effects in respiratory models by reducing neutrophilic inflammation, oxidative damage, and cytokine production [66,67].

Therefore, native Brazilian fruits may represent a promising dietary strategy to support respiratory health, particularly in populations exposed to high levels of environmental pollutants. However, direct evidence from experimental and clinical studies specifically targeting airway inflammation remains limited. Future research should investigate the effects of these fruits in respiratory disease models, including COPD and asthma, to better understand their potential role in modulating airway inflammation and improving pulmonary outcomes.

## 6. Inflammation in the Context of Environmental Challenges

Importantly, environmental stressors such as climate change, rising global temperatures, and air pollution are increasingly recognized as contributors to systemic inflammatory states. Sugden et al. [5] explored this relationship, showing that these exposures intensify oxidative stress and inflammatory responses, thereby increasing vulnerability to psychiatric conditions.

In parallel, the adoption of Western dietary patterns further fuels systemic inflammation, while plant-based diets emerge as a dual strategy to mitigate both neuroinflammatory processes and climate change. Diets centered on minimally processed plant foods are associated with lower levels of circulating pro-inflammatory cytokines and, at the same time, contribute to a reduction in greenhouse gas emissions, highlighting the intersection between individual mental health and planetary health [5,6,57,61].

Thus, when addressing neuroinflammation, it is essential to consider the combined influence of dietary patterns and environmental exposures. Strategies that promote predominantly plant-based diets, coupled with the reduction in ultra-processed food consumption and the implementation of policies to mitigate air pollution and climate change, can have synergistic effects in reducing systemic and central inflammation, ultimately benefiting mental health [5,68].

The cultivation and consumption of native Brazilian fruits, rich in bioactive compounds with antioxidant and anti-inflammatory properties, emerge as a promising approach. On top of to modulating oxidative and inflammatory pathways associated with mental well-being, these crops contribute to biodiversity preservation and the maintenance of balanced ecosystems, as they are based on low-impact production systems adapted to the local environment and aligned with the conservation of natural resources [68].

## 7. A Counterpoint to the Most Studied Fruits in the Global Scenario

The findings of this review highlight the antioxidant, anti-inflammatory and neuroprotective potential of native Brazilian fruits, positioning them as relevant yet still underrepresented counterparts to globally consolidated fruits such as blueberries, strawberries and pomegranates. These widely studied fruits are rich in polyphenols and have demonstrated the ability to modulate inflammatory and oxidative biomarkers in both experimental and clinical settings [69,70]. In this sense, the functional properties reported for Brazilian native species challenge the current asymmetry in the scientific literature, which remains disproportionately centered on temperate fruits despite the comparable phytochemical richness and biological potential observed in tropical biodiversity [14].

The integration of native Brazilian fruits into established dietary patterns provides a relevant translational framework for understanding their potential impact on human health. Several of the bioactive compounds identified in this review, particularly anthocyanins, flavonols, carotenoids, and monounsaturated fatty acids, are also central components of the Mediterranean diet, one of the most extensively studied anti-inflammatory dietary models. Evidence from the PREDIMED (Prevención con Dieta Mediterránea) trial demonstrates that greater intake of dietary polyphenols, characteristic of Mediterranean dietary patterns rich in plant-based foods such as fruits, is associated with a lower risk of all-cause mortality in individuals at high cardiovascular risk [71]. Similarities in phytochemical composition between Mediterranean fruits and native Brazilian species such as jaboticaba and cambuci reinforce the plausibility that their polyphenols may confer comparable physiological benefits [72].

Within the Brazilian context, the inclusion of these fruits aligns closely with the principles of the traditional Brazilian dietary pattern and the “Guia Alimentar para a População Brasileira” (Dietary Guidelines for the Brazilian Population), which emphasizes minimally processed plant foods, biodiversity, and cultural culinary practices [73]. Many native fruits historically constituted essential components of regional food systems but remain underutilized in modern diets despite their nutrient density and functional properties, a process that mirrors broader patterns of dietary homogenization and erosion of food biodiversity [74].

This cultural gap is starkly reflected in the current dietary patterns of the Brazilian population. According to data from the National Dietary Survey [75], of the ten most consumed fruits in the country, only two (açaí and pineapple) are native to Brazil. This dominance of exotic species in the national diet underscores a profound disconnection between the country’s vast biological heritage and its food habits. By highlighting the functional potential of less explored species, this review aims to provide the evidentiary basis needed to reverse this trend, encouraging the reintegration of native biodiversity into daily consumption as a strategy to combat nutritional monotony and strengthen local food sovereignty.

On top of that, diets rich in diverse plant bioactives, whether Mediterranean or hybrid models, are associated with improved gut microbiota profiles, lower systemic inflammation, and enhanced metabolic and neurocognitive outcomes [70,71]. Given their phytochemical richness, native Brazilian fruits may therefore function as strategic components within evidence-based dietary patterns aimed at mitigating inflammatory processes while simultaneously supporting food biodiversity and cultural sustainability in Brazil. Although native Brazilian fruits still lack large-scale randomized clinical trials, in vivo studies with guaraná and açai have already demonstrated behavioral improvement and reduction in inflammatory markers in the central nervous system, pointing to a promising translational pathway for mental health research [14].

It is worth noting that the beneficial effects of native Brazilian fruits depend on agronomic and technological factors, including cultivation conditions, ripening stage, plant part used (pulp, peel, seeds and even branches) and preparation methods, all of which significantly influence the bioavailability and biological efficacy of bioactive compounds. Therefore, methodological standardization across experimental and clinical studies is essential to consolidate robust evidence on the role of these fruits in inflammation-related disorders and mental health outcomes, enabling their consistent integration into dietary recommendations and public health strategies.

The scientific asymmetry between well-characterized temperate fruits and the ‘under-researched’ tropical species is not merely a data gap, but a barrier to global nutritional security. While global dietary guidelines often rely on a narrow set of commercial species, the chemical diversity of the Brazilian flora offers a vast, untapped repertoire of bioactive compounds that could redefine public health strategies. Aligning these findings with the “Guia Alimentar para a População Brasileira”, the promotion of native fruits transcends individual nutrition; it fosters a sustainable food system based on biodiversity, seasonality, and regional identity.

Strengthening the value chain of native fruits creates a powerful incentive for conservation. By establishing these species as high-value functional ingredients, we provide economic justification for the preservation of biomes like the Cerrado, Atlantic Forest, and the Amazon, shifting the paradigm from land clearing to sustainable harvesting. Furthermore, integrating these fruits into public health policies can mitigate the impact of the ‘nutrition transition’ in South America, offering culturally relevant and nutrient-dense alternatives to ultra-processed foods. Ultimately, addressing this scientific gap is a step toward ‘nutritional decolonization,’ where the valorization of local biodiversity becomes a primary tool for both ecological sustainability and the promotion of metabolic health on a global scale.

## 8. Experimental Evidence and Study Models

In vivo studies accounted for the majority of the selected articles. Among the 25 experimental studies included, 11 were conducted exclusively in vivo, 6 exclusively in vitro, 7 combined in vivo and in vitro approaches, and 1 combined ex vivo and in vitro analyses.

With respect to study populations, animal models were used in 24 of the 25 studies. Mice were the predominant experimental model, employed in 20 studies, either in vivo, in vitro (murine-derived cells), or in combined designs. One study used female rabbits as the in vivo model [18], and one study employed *Drosophila melanogaster* as an alternative in vivo model [22]. Human participation was reported in only one study, which adopted an ex vivo design using samples from healthy female volunteers [38]. In vitro experiments were primarily conducted using murine-derived immune, neural, or fibroblast cell lines, reinforcing the strong reliance on murine systems across experimental approaches.

Regarding the plant parts analyzed, fruit-derived matrices predominated, being investigated in 19 studies. Within this group, peels were the most frequently analyzed fruit fraction, reported in 6 studies, particularly in jaboticaba, marolo, and cambuci. Seeds and seed-derived products (including oils and hydroalcoholic extracts) were evaluated in 7 studies, mainly involving açai, guarana, cutia fruit, jucá, and macaúba. Pulp-based preparations (pulp, juice, or pulp with seeds) were analyzed in 5 studies, while whole-fruit preparations, including lyophilized whole fruit or combined pulp-and-peel extracts, appeared in 4 studies.

In addition to edible fruit parts, non-fruit plant tissues were assessed in 5 studies, including leaves and branches, notably in *Eugenia* species, jaboticaba trees, and pinhão-manso. These studies highlight the exploration of underutilized plant parts as alternative sources of bioactive compounds with anti-inflammatory potential.

Overall, the experimental evidence supporting the anti-inflammatory effects of native Brazilian fruits is overwhelmingly derived from preclinical animal models, particularly mice, and from fruit-based matrices, with peels and seeds emerging as the most frequently studied plant parts. Human-based evidence remains scarce, underscoring the need for future translational and clinical studies to validate these findings.

## 9. Limitations, Bioavailability and Therapeutic Potential

Despite promising anti-inflammatory and antioxidant effects observed across studies, several methodological and biological limitations must be considered when interpreting these findings. A major constraint is the predominance of in vitro and animal models, which restricts the extrapolation of results to human physiology. The scarcity of well-designed human clinical trials further limits the ability to establish causal relationships and to translate these findings into evidence-based dietary recommendations. Only a minority of studies assessed bioavailability, absorption, or biotransformation of the bioactive compounds, leaving a substantial gap in understanding how these phytochemicals behave in vivo. This gap is particularly relevant because the interaction between polyphenols and the gut microbiota, a central determinant of phenolic metabolism and production of bioactive metabolites, remains largely unexplored in the context of native Brazilian fruits. Compounds such as polyphenols and carotenoids undergo extensive metabolic transformations during digestion, including enzymatic modification and phase II conjugation, which may substantially alter their biological activity and systemic availability [52].

Another critical limitation is the pronounced heterogeneity of the samples investigated. Native Brazilian fruits exhibit wide variability in their phytochemical composition depending on species, maturity stage, geographic origin, cultivation conditions, and post-harvest handling [32,49]. Such variability directly influences the concentration and profile of bioactive compounds. Moreover, processing methods, including whether fruits were used fresh, lyophilized, fermented, or transformed into extracts, significantly modulate compound stability and bioaccessibility. Fermentation can increase the release and bioaccessibility of polyphenols, whereas thermal processing may degrade heat-sensitive molecules [37,76,77]. Differences in dosage, duration of exposure, inflammatory stimuli, and experimental models (cell lines, rodents, or isolated tissues) further complicate direct comparison between studies. Importantly, many experimental studies employ concentrations of isolated compounds or extracts that exceed levels achievable through habitual dietary intake, highlighting a gap between experimental conditions and real-world consumption [6].

Methodological differences also influence the interpretation of antioxidant outcomes. As highlighted by Vuolo et al. (2019) [50], in vitro assays such as DPPH, FRAP, and ABTS differ substantially in the radical species employed, reaction kinetics, and sensitivity to antioxidant subclasses. Such inconsistencies contribute to variability across studies and should be considered when comparing the antioxidant potential of different native fruits.

The lack of standardized extraction methods, quantification protocols, and dose normalization also contributes to inconsistent reporting. Variability in plant parts used (pulp, peel, seed) and the absence of detailed phytochemical characterization in some studies add another layer of complexity. Furthermore, matrix effects and interactions with other dietary components can influence the digestion, absorption, and bioefficacy of these compounds, reinforcing the importance of interpreting results within the context of whole foods rather than isolated extracts [6]. Together, these factors highlight the need for reproducible methodologies and precise analytical approaches to enable more reliable comparisons and meta-analytical synthesis.

Even with these limitations, the evidence consistently demonstrates the therapeutic potential of these fruits. Across models, native Brazilian species were able to modulate key inflammatory pathways, particularly through the inhibition of NF-κB and NLRP3 inflammasome signaling, reduce levels of pro-inflammatory cytokines, enhance endogenous antioxidant defenses, and attenuate both systemic and, in a smaller subset of studies, neuroinflammatory markers. However, caution is warranted when extrapolating these findings to clinical or public health contexts, given the limitations discussed. Future research should prioritize well-controlled human intervention studies, standardized dosing strategies, and integrative approaches that account for bioavailability and food matrix interactions. These mechanistic effects nonetheless support the incorporation of native fruits into anti-inflammatory dietary strategies.

## 10. Final Considerations

Although the available findings are promising, important translational challenges remain. Current evidence is still predominantly preclinical, with limited applicability to humans due to the scarcity of controlled clinical trials. In addition, substantial variability in extraction methods, phytochemical characterization, dosage protocols, and experimental designs limits direct comparison among studies and compromises standardization of findings.

Another critical gap involves the limited understanding of bioavailability, metabolism, and biotransformation of these compounds, since high in vitro antioxidant capacity does not necessarily translate into physiological effects after digestion and absorption.

Future research should prioritize well-designed clinical studies involving different populations, standardized extracts, and integrated evaluation of inflammatory, metabolic, and mental health outcomes. Investigating synergistic interactions between native fruits and broader dietary patterns may also contribute to a more comprehensive understanding of their role in chronic disease prevention.

In this context, Brazilian biodiversity represents not only a valuable source of bioactive compounds but also an opportunity to integrate nutrition, sustainability, public health, and cultural identity through the promotion of native food resources. Advancing these findings from experimental evidence to practical nutritional applications will depend on greater methodological standardization, translational investigation, and long-term human studies.

## 11. Conclusions

Brazilian fruits are important sources of bioactive compounds, particularly polyphenols, carotenoids, terpenoids, and bioactive fatty acids. Most evidence from their beneficial effects derives from cellular and animal models, in which fruit-derived extracts were consistently associated with the attenuation of oxidative stress, reduction in inflammatory mediators, and modulation of pathways involved in chronic inflammatory and neuroinflammatory conditions. Modulation of redox-sensitive inflammatory pathways, especially those involving NF-κB signaling, cytokine production, oxidative biomarkers, and endogenous antioxidant defenses, represented the most consistent findings. These effects were also accompanied by reduced leukocyte infiltration, tissue damage, and neuroinflammatory markers, including attenuation of microglial and astrocytic activation. Therefore, native Brazilian fruits are promising dietary sources of bioactive compounds for modulating oxidative and inflammatory responses at both systemic and neuroimmune levels, lending support to their potential relevance in health promotion and for the industry of functional foods.

## Figures and Tables

**Table 1:** Index of common and scientific names of native Brazilian fruits with an impact on oxidative stress and inflammation.

Image	Common Name	Scientific Name
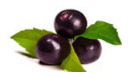	Açai	*Euterpe oleracea* Mart.
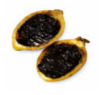	Blackberry Jam	*Randia formosa* (Jacq.) K. Schum
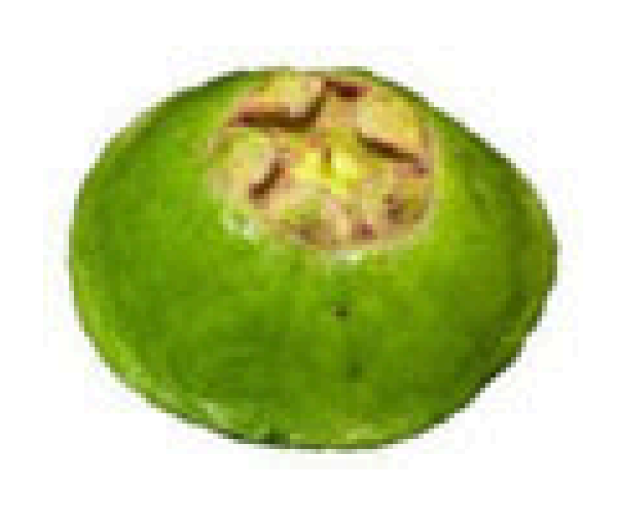	Cambuci	*Campomanesia phaea* (O. Berg) Landrum
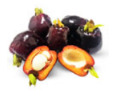	Cereja-do-Rio-Grande	*Eugenia involucrata* DC.
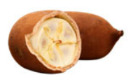	Cupuaçu/Cupuassu	*Theobroma grandiflorum* (Willd. ex Spreng.) K. Schum.
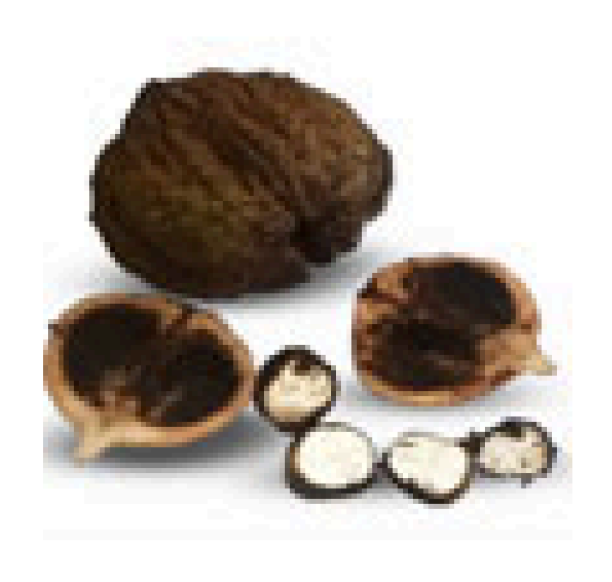	Cutia	*Carpotroche brasiliensis* (Raddi) A. Gray
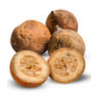	Genipapo	*Genipa americana* L.
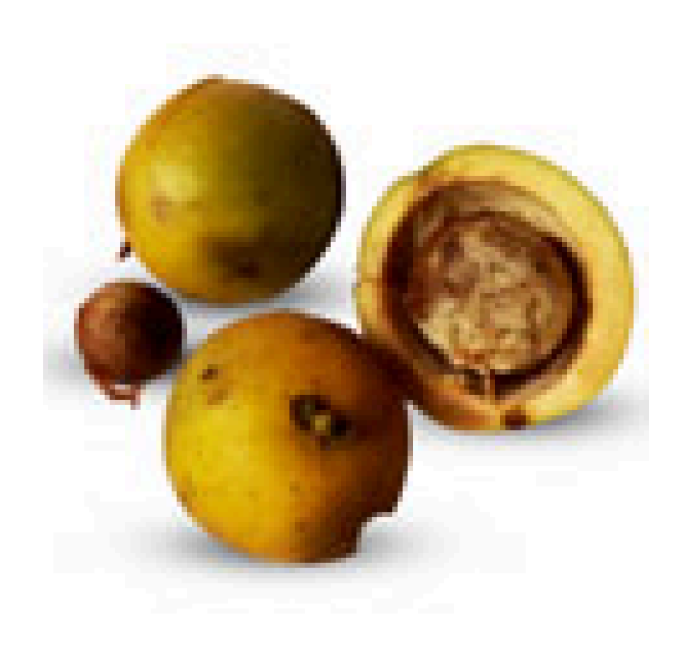	Goiabão	*Eugenia leitonii* D.Legrand
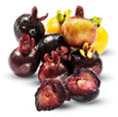	Grumixama	*Eugenia brasiliensis* Lam.
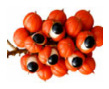	Guarana	*Paullinia cupana* var. *sorbilis* Mart.
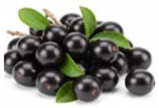	Jaboticaba	*Plinia cauliflora* (Mart.) Kausel
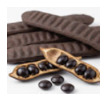	Jucá	*Libidibia ferrea* (Mart. ex Tul.) L.P.Queiroz
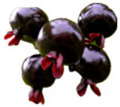	Juçara	*Euterpe edulis* Mart.
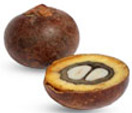	Macaúba	*Acrocomia aculeata* (Jacq.) Lodd. ex Mart.
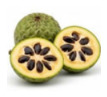	Marolo	*Annona crassiflora* Mart.
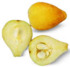	Pêssego-do-Mato	*Eugenia myrcianthes* Nied
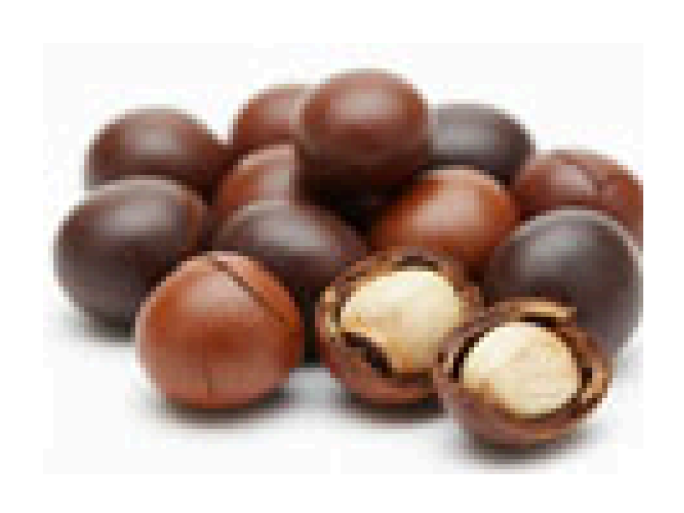	Pinhão-manso	*Jatropha curcas* L.
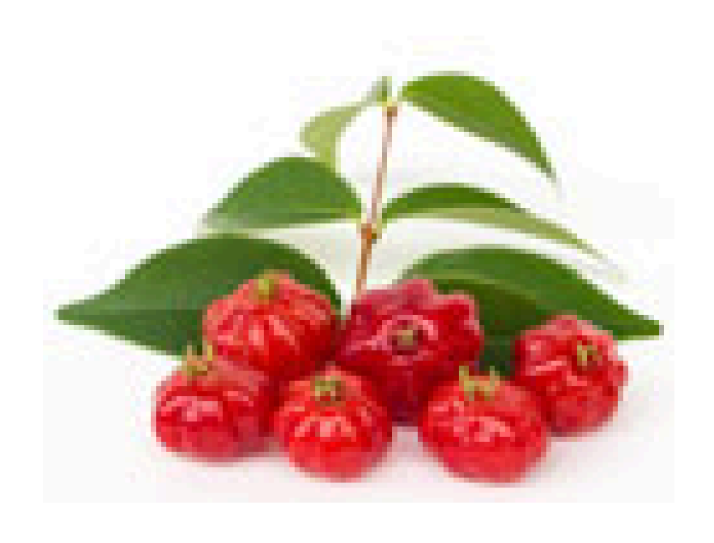	Pitanga	*Eugenia uniflora* L.
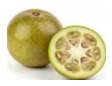	Tomatinho-do-mato	*Solanum diploconos* (Mart.) Bohs

**Table 2:** Summary of individual experimental studies evaluating Brazilian native fruits and their redox- and inflammation-related outcomes. Each row represents a distinct study.

Common Name (Scientific Name)	Study	Study Design	Study Sample	Analyzed Part	Dosage	Phytochemical Composition	Toxicity	Inflammatory Mechanisms	Main Findings
Jaboticaba (*Plinia cauliflora*)	[15]	Experimental in vivo study	Swiss albino mice (*Mus musculus*)	Peel: Hydro-ethanolic extract (PcE)	12.5, 25, 50, and 100 mg/kg orally	Phenolics: tannins and phenolic acids (gallic and ellagic acid)	No toxicity (2000 mg/kg)	↓ free radicals, leukocyte migrations abdominal pain	PcE showed potent antioxidant activity, reduced inflammation, had analgesic effects, and improved glucose and lipid markers in diabetic mice.
	[16]	Experimental in vitro and in vivo study	Swiss mice	Leaves & branches: Ethanolic Extract (EELPc & EEBPc)	In vitro: 31.25, 62.5, and 125 μg/mL (DMSO 0.5%) In vivo: 0.125, 0.25, and 0.5 mg/ear by topic essay	Phenolics: flavonoids, (quercetin and myricetin), phenolic acids (gallic and ellagic acids) and tannins	In vivo: n.a. In vitro: No cytotoxicity (MTT ≥ 70%), except at 125 μg/mL (EEL)	↓ edema, consumption of DPPH	EEPc showed high antioxidant and anti-inflammatory potential, highlighting under-used plant parts for bioactive applications.
	[17]	Experimental in vivo study	C57BL/6J Mice	Whole fruit lyophilized: polyphenol extract (PEJ)	50 and 100 GAE/kg orally	Phenolics: flavonoids (anthocyanins, quercetin and rutin) & phenolic acids (gallic and ellagic acids)	n.a.	↓ NLRP3, ASC, IL-1β	PEJ exhibited strong anti-inflammatory and metabolic effects, improving intestinal inflammation, metabolic parameters and gut microbiota in obesity contexts.
	[18]	Experimental in vivo study	New Zealand female rabbits	Peels: Hydroethanolic extract (EEPC)	75 and 150 mg/kg intravenously	Phenolics: flavonoids (anthocyanins, quercetin, myricetin), phenolic acids (gallic acid, ellagic acid) and tannins (gallotannins, ellagitannins)	n.a.	↓ NT, MDA	EEPC prevented heart failure by enhancing antioxidant defense and preventing cardiac damage, suggesting cardioprotective adjuvant potential.
	[19]	Experimental in vivo study	Male Swiss inbred strain mice	Peel: Freeze-dried jaboticaba peel powder (FDJPP)	1, 2 or 4% *w*/*w* in fed	Terpenes & Phenolics: flavonoids (quercetin) and phenolic Acids (ellagic acid)	n.a.	↓ IL-1b, IL-6, IkB-a	FDJPP reduced insulin resistance and hepatic inflammation induced by a high-fat diet, suggesting protection against obesity-associated insulin resistance.
Guarana (*Paullinia cupana*)	[20]	Experimental in vitro study	Mouse (Embryonic fibroblast NIH-3T3 cell)	Seed powder	0.5, 1, 5, 10 and 20 mg/mL of extract	Caffeine; Theobromine & Phenolics: flavonoids and tannins	No relevant cytotoxicity; slight viability decrease at ≥10 mg/mL	↓ NO, ROS, ONOO ↑ SOD	Significantly reduced cell mortality, lipid peroxidation, DNA damage (especially at doses ≤5 mg/mL), and intracellular oxidative stress. Increased SOD activity, suggesting an adaptive antioxidant response. The protective effects were dose-dependent, with best performance between 0.5–5 mg/mL. Higher doses (10–20 mg/mL) showed a hormetic response, with partial loss of cell protection.
	[21]	Experimental in vitro study	Brain and cerebellum cells from mice	Seed powder: Hydroalcoholic extract	10, 30, 100 and 300 µg/mL of extract	Caffeine; Theobromine & Phenolics: flavonoids (catechins)	No cytotoxicity detected (10, 30, 100 and 300 µg/mL)	↓ ROS ↑ CAT	It showed no toxicity at any of the concentrations tested, protected brain and cerebellar cells and increased cell viability. The neuroprotective effect was most pronounced at concentrations of 100 and 300 µg/mL
	[22]	Experimental in vivo and in vitro study	In vivo: Fruit Flies (Drosophila melanogaster Canton-S wild-type) In vitro: SH-SY5Y neuronal-like cells	In vivo: Seed powder In vitro: Seed powder hydroalcoholic extract	In vivo: 1, 5, 10, and 20 mg/mL of extract In vitro: 50–1000 μg/mL of extract	Caffeine, Theobromine & Phenolics: flavonoids (catechins)	In vivo: No toxicity at 1–10 mg/mL; Reduced viability at 20 mg/mL In vitro: No cytotoxicity ≤100 μg/mL	↓ IL-1β, IL-6, TNF-α, IFN-γ, ROS ↑ IL-10	In vivo: it improved locomotor performance, prevented oxidative stress, normalized inflammatory parameters, and protected against the neurobehavioral toxicity of MeHg In vitro: It increased cell viability and protected neural cells against MeHg toxicity.
	[23]	Experimental in vivo	Wistar male albino rats, *Rattus norvegicus*	Seed powder	300 mg/kg/day orally	Caffeine & Phenolics: flavonoids (catechins and epicatechins)	n.a.	↓ TNF, ROS ↑ SOD	Prevention of liver necrosis, protein carbonylation, glutathione depletion, and improvement of total antioxidant capacity
Açai (*Euterpe oleracea*)	[24]	Experimental in vivo study	Male mice of the C57BL/6	Seed: Hydroalcoholic extract (ASE)	300 mg/kg/day orally	Phenolics: flavonoids (proanthocyanidins)	n.a.	↓ RAS, MDA, TNF-α, IL-6 ↑ SOD	Prevention of body weight gain, adipocyte hypertrophy and increase in visceral adipose tissue, dyslipidemia, hyperglycemia and insulin resistance
	[25]	Experimental in vivo study	Male Wistar	Seed: Hydroalcoholic extract (ASE)	200 mg/kg/day orally	Phenolics: flavonoids (proanthocyanidins)	n.a.	↓ CD45, IL-6, TNF-α, MCP-1, NADPH, ROS, TGF-β1 ↑ SOD, CAT, GPx	ASE consumption led to structural and functional renal protection, especially in hypertensive diabetic animals, reduction in renal apoptosis, significant reduction in inflammation and reduction in oxidative stress.
Marolo (*Annona crassiflora*)	[26]	Experimental in vivo and in vitro study	In vivo: Male C57BL/6/J/UFU mice In vitro: BMDM cells	Peel: polyphenol-enriched fraction (EtOAc)	In vivo: 0.1, 0.3, 1.0 μg mL^−1^ of extract In vitro: 10, 30, 100 mg kg^−1^ orally	Phenolics: flavonoids (catechin, quercetin, rutin) and phenolic acids (gallic acid)	In vivo: n.a. In vitro: No cytotoxicity (MTT, NIH/3T3 cells)	↓ IL-6, NO, spontaneous nociception, edema	Support the antinociceptive and anti-inflammatory effects
	[27]	Experimental in vitro and in vivo study	Fibroblasts (L292) and keratinocytes (HaCaT)	Peel: fraction rich in phenolic compounds (PCAc)	4% of PCAc of extract	Phenolics: flavonoids (quercetin and catechin) and phenolic acid (gallic acid)	In vivo: n.a. In vitro: No cytotoxicity (≤100 μg/mL; proliferative effect >50 μg/mL)	↓ neutrophil and macrophage activity ↑ MMP-9, MMP-2	PCAc promoted fibroblast proliferation, wound-diameter reduction, collagen improvement and antioxidant activity–potential for healing formulations.
Pinhão manso (*Jatropha curcas*)	[12]	Experimental in vitro study	Glial cells from the cerebral cortex of neonate Wistar rats	Leaves: Methanolic Extract (MEJ)	0.1–50,000 μg mL^−1^ of extract and fractions: 0.1 μg mL^−1^	Steroids; Terpenoids (saponins) & Phenolics: flavonoids (flavanones and anthocyanidins) and xanthones	No cytotoxicity (0.1–1000 μg mL^−1^)	↓ GFAP, Iba1 expressions; ↓ NF-kB and NO production	MEJc and its fractions modulate the inflammatory response of astrocytes and microglia to LPS, potentially serving as a therapeutic strategy for neuroinflammatory diseases.
Tomatinho do mato (*Solanum diploconos*)	[28]	Experimental in vitro study	N/A	Pulp & Peel: freeze-dried Whole Fruit: Ethanolic Extract	Pulp & Peel: 0.5–1 g (freeze-dried) Whole fruit: 0.05–1000 µg/mL of extract (ethanol, 1:10)	Phenolic compounds; Carotenoids; Tocopherols & Ascorbic acid	n.a.	↓ ROS and RNS	Fruits may be seen as a promising source of bioactive compounds with high antioxidant potential against the most physiologically relevant ROS and RNS
Jucá/Pau-ferro (*Libidibia ferrea*)	[29]	Experimental in vivo and in vitro study	Male Swiss mice	Seeds: Protease inhibitor purified (LfTI)	In vitro: 5, 50 and 250 μg/mL of extract In vivo: 0.6, 3 and 15 mg/kg subcutaneously	Natural compounds of the fruit, which are not specified	In vivo: n.a. In vitro: No cytotoxicity (5, 50 and 250 μg/mL)	↓ H_2_O_2_, NO, vascular permeability, paw edema	LfTI has anti-inflammatory and neurogenic pain effects, and these effects might be associated with the inhibition of inflammatory proteases
Cambuci (*Campomanesia phaea*)	[30]	Experimental in vivo study	Male C57BL/6J mice	Pulp: Polyphenol extract (CBC)	36 or 74 mg/kg orally	Phenolics: flavonoids (proanthocyanidins, catechin, quercetin, myricetin and rutin) and phenolic Acids (ellagic and gallic acid)	n.a.	↓ IL-6, TNF-α, of SAPK/JNK phosphorylated, NF-κB	CBC reduced body weight gain, inflammation, hepatic steatosis, hyperglycemia, glucose intolerance, and insulin resistance in liver and skeletal muscle of obese mice
Goiabão (*Eugenia leitonii*); Cereja do Rio Grande (*Eugenia involucrata*); Grumixama (*Eugenia brasiliensis*); Pêssego-do-mato (*Eugenia myrcianthes*)	[31]	Experimental in vitro and in vivo study	Male Balb/c albino mice	Seeds, pulp & leaves: ethanolic extracts (EE)	2 g of the lyophilized material for each EE orally	Phenolics: flavonoids (epicatechin) and phenolic acids (gallic acid)	n.a.	↓ ROS, neutrophil influx	Leaves of these native fruits showed the highest antioxidant and anti-inflammatory activities, suggesting use as food additives or functional-food ingredients.
Genipapo (*Genipa americana*)	[32]	Experimental in vitro study	N/A	Whole fruit: Genipin-NADES extract	2 μL (1 mg/mL) of extract	Genipin	No toxicity (based on literature data)	AChE and BChE inhibition	Genipin extract showed antioxidant activity and protein-crosslinking capacity, indicating therapeutic potential for neuro-degenerative disorders and use in nutraceutical/pharmaceutical formulations.
Cutia fruit (*Carpotroche brasiliensis*)	[33]	Experimental in vivo study	Mice Wistar rats and Swiss albino mice	Seed: oil	10–500 mg/kg orally	Fatty acids: MUFA	No mortality ≤300 mg/kg (mild lethargy); 30% mortality at 500 mg/kg	↓ paw edema, hyperalgesia, pain	Acid fraction showed significant anti-inflammatory and antinociceptive activity in acute models, with no effect in chronic inflammation models.
Macaúba (*Acrocomia aculeata*)	[34]	Experimental in vivo study	Black male mice C57Bl/6	Pulp: oil (HFM)	40 g/kg (4%) of oil	Carotenoids; Tocopherols & Fatty acids: MUFA (oleic acid)	n.a.	↑ SOD ↓ MDA, Serum TAC, NF-κB, PPAR-γ	Macaúba pulp oil prevents oxidative stress, inflammation and adipogenesis, increasing antioxidant capacity and showing potential against high-fat-diet (HFD) metabolic changes.
Blackberry Jam (*Randia formosa*)	[35]	Experimental in vitro study	N/A	Pulp & Seeds: Hydroalcoholic extract (BJFp)	0.2 g/mL (20% m/v) of extract	Lactone & Phenolics: coumarin and flavonoids	n.a.	↓ LPO, CP, AChE, BChE ↑ SOD, CAT	BJFp protected against oxidative stress; further in vivo investigation needed.
Juçara (*Euterpe edulis*)	[36]	Experimental in vitro study	J774 murine macrophage cells (ATCC TIB-67)	Pulp: crude extract and fractions	1–100 µg/mL of extract	Fatty acids: MUFA (oleic and linolenic) & Phenolics: flavonoids and phenolic acids	Low cytotoxicity: crude extract, hexane and ethyl acetate non-cytotoxic (1–100 μg/mL); dichloromethane and butanol show cytotoxicity at 100 μg/mL	↓ NO, IL-12, IL-6, IFN-γ	Anti-inflammatory effect was observed depending on the fraction, especially with hexane and dichloromethane, while more polar fractions (ethyl acetate and butanol) showed a weaker effect
Cupuaçu/cupuassu (*Theobroma grandiflorum*)	[37]	Experimental in vivo study	Male C57BL/6 mice	Pulp: unfermented and fermented juice	120 mg/mL orally	Unfermented—Organic Esters & Phenolics: phenolic acids, alcoholic phenols Fermented—Alkaloids & Phenolics: phenolic acids and flavonoids	n.a.	↓ systemic inflammation	Fermentation altered the profile of bioactive compounds, enhancing the biological activity of the juice. Fermented juice more markedly reduced the clinical severity of systemic inflammation, decreased leukocyte migration and attenuated inflammation-related alterations in organs.
Pitanga (*Eugenia uniflora*)	[38]	Experimental ex vivo and in vitro	Ex vivo: Healthy female volunteers In vitro: HGF-1 cells	Pulp: Juice	100 mL (35% pulp) of pitanga juice	Terpenes & Phenolics: flavonoids (anthocyanin)	Ex vivo: n.a. In vitro: No cytotoxicity (10 min pre-treatment or 6 h co-treatment with PG-LPS)	↓ IL-8; CXCL8	Reduction in LPS-induced inflammatory activation in *P. gingivalis*. The compounds did not show a preventive effect, but they did show a therapeutic anti-inflammatory effect when incubated concurrently with the inflammatory stimulus. The study demonstrates for the first time the anti-inflammatory potential of oxidoselin-1,3,7(11)-trien-8-one.

Captions: N/A: Not applicable; n.a.: Not accessed. Note: The units reported correspond to experimental doses or extract concentrations used in the original studies (e.g., mg/kg, μg/mL, %, GAE/kg), and not to bioactive compound contents expressed per 100 g of fresh or dry food weight. ↑ and ↓ means increase and decrease, respectively.

**Table 3:** Bioactive compounds and antioxidant activities of native Brazilian fruits. Each row corresponds to a specific study and its respective analyzed parts.

Fruit	Study	Analysed Part (Form)	Phytochemical Compounds ^1^	Antioxidant Activity ^1^
Jaboticaba (*Plinia cauliflora*)	[15]	Peels (hydro-ethanolic extract)	n.a.	DPPH: 32.29 ± 0.2% inhibition (0.25 mg/mL); 58.03 ± 0.10% inhibition (0.5 mg/mL); 92.68 ± 0.40% inhibition (1 mg/mL) ^2^
	[16]	Branches (ethanolic extract)	n.a.	DPPH: 1.45 ± 0.02 µg/mL (IC_50_) PM: 209.18 ± 15.2% of rutin; 80.44 ± 5.84% of quercetin BCB: 71.47 ± 5.64% inhibition (38.5 μg/mL)
		Leaves (ethanolic extract)	n.a.	DPPH: 1.43 ± 0.08 µg/mL(IC_50_) PM: 261.74 ± 33.5% of rutin; 97.44 ± 12.9% of quercetin BCB: 62.50 ± 4.18% inhibition (38.5 μg/mL)
	[18]	Peels (hydroethanolic extract)	n.a.	NT & MDA: ≈80% reversed SOD & CAT: ≈50% loss reversed Lipid hydroperoxides: >400% increase reversed
Guarana (*Paullinia cupana*)	[20]	Seeds (powder hydroethanolic extract)	Caffeine: 12.24 mg/g Theobromine: 6.73 mg/g Total cathequins: 4.34 mg/g Condensed tannins: 16 mg/g	n.a.
	[21]	Seeds (powder hydroethanolic extract)	Caffeine: 12.24 mg/g Theobromine: 6.73 mg/g Total cathequins: 4.34 mg/g	n.a.
	[22]	Seeds (powder hydroethanolic extract)	Caffeine: 12.24 mg/g d.b. Theobromine: 6.73 mg/g d.b. Total catechins: 4.34 mg/g d.b.	n.a.
	[23]	Seeds (powder)	Total polyphenols: 4459.41 ± 88.23 mg GAE/100 g	ABTS: 386.87 µM TE/g DPPH: 146.6 g guarana/g DPPH (IC_50_) FRAP: 224.79 ± 0.68 µM TE/g ORAC: 221.10 ± 12.53 µM TE/g
Açai (*Euterpe oleracea* Mart.)	[39]	Pulp (lyophilized)	Total polyphenols: 77.51 ± 0.54 mg GAE/g d.b. Anthocyanin: 693.85 ± 2.03 mg/100 g d.b.	DPPH: 289.08 ± 6.83 μmol TE/g d.b. ABTS: 385.92 ± 8.90 μmol TE/g d.b. FRAP: 500.35 ± 8.66 Ferrous Sulfate μm/g d.b.
	[40]	Seeds (methanolic extract)	n.a.	DPPH: 16.95 ± 0.18 mg/L (EC_50_) TEAC: 3835.44 ± 73.50 μmol Trolox/g ORAC: 4082.16 ± 58.55 μmol Trolox/g
Marolo (*Annona crassiflora*)	[41]	Pulp (hydroethanolic extract)	Total polyphenols: 5.89 ± 0.11 mg GAE/g d.b. Flavonoid: 2.53 ± 0.51 mg QE/g	DPPH: 182.54 ± 7.3 µg/mL (EC_50_) ABTS: 94.66 ± 1.9 µM TE/g
		Pulp (ethyl acetate extract)	Total polyphenols: 33.16 ± 1.1 mg GAE/g d.b. Flavonoid: 70.55 ± 0.5 mg QE/g	DPPH: EC_50_: 57.80 ± 3.9 µg/mL (EC_50_) ABTS: 192.61 ± 2.8 µM TE/g
Pinhão manso (*Jatropha curcas*)	[42]	Leaves (powder)	Phenolic acid 1.29 ± 0.053 mg.g^−1^ Flavonoids 0.540 ± 0.011 mg.g^−1^ Tannins 0.870 ± 0.16 mg.g^−1^	DPPH: 13.53 ± 0.22 mg.g^−1^
	[43]	Shell (ethanolic extract, 95%)	Total polyphenols: 536.33 ± 8.62 μg GAEs/mg	DPPH: 13.63 ± 0.15 μg/mL ABTS: 6.75 ± 0.51 μg/mL
Tomatinho do mato (*Solanum diploconos*)	[28]	Pulp (freeze-dried)	Carotenoids: 44 ± 6 μg/g d.b. Total polyphenols: 1429 ± 36 μg/g d.b.	n.a.
		Peels (freeze-dried)	Carotenoids: 25 ± 5 μg/g d.b. Total polyphenols: 1085 ± 108 μg/g d.b.	
		Whole fruit (ethanolic extract)	Carotenoids: 27 ± 6 μg/g d.b. Total polyphenols: 3817 ± 246 μg/g d.b.	Scavenging capacity against all tested ROS and RNS (IC_50_ = 14–461 μg/mL)
Jucá/Pau-ferro (*Libidibia ferrea*)	[44]	Leaves (hydroethanolic extract)	Total polyphenols: 554.94 ± 1.24 mg GAE/g Total Flavonoids: 32.86 ± 0.10 mg QE/g Tannins: 472.29 ± 1.52 mg TAE/g	DPPH: 9.44 ± 0.84 µg/mL (IC_50_) ABTS: 12.70 ± 0.14 µg/mL (IC_50_)
		Seeds (methanolic extract)	Total polyphenols: 506.82 ± 1.14 mg GAE/g Total Flavonoids: 18.22 ± 0.80 mg QE/g Tannins: 476.63 ± 0.86 mg TAE/g	DPPH: 2.39 ± 0.10 µg/mL (IC_50_) ABTS: 7.02 ± 0.09 µg/mL (IC_50_)
Cambuci (*Campomanesia phaea*)	[30]	Pulp (polyphenol extract—17.5 mL of water)	Total polyphenols: 4.45 mg GAE/mL Proanthocyanidins: 0.23 ± 0.01 mg Cya-chlor/mL Ellagic acid: 2545 ± 95 μg/100 mL Quercetin pentoside: 728 ± 50 μg/100 mL	n.a.
		Pulp (polyphenol extract—35 mL of water)	Total polyphenols: 9.20 mg GAE/mL Proanthocyanidins: 0.54 ± 0.03 mg Cya-chlor/mL Ellagic acid: 4333 ± 34 μg/100 mL Quercetin pentoside: 1469 ± 31 μg/100 mL	
Goiabão (*Eugenia leitonii*)	[31]	Leaves (ethanolic extract)	Total polyphenols: 88.62 ± 2.43 mg GAE/mL Epicatechin: 31.25% ^3^ Gallic acid: 13.36% ^3^	DPPH: 112.38 ± 1.8 μg/mL (EC_50_) BCB: 3.01 ± 0.5 μmol Trolox/g ORAC: 417.71 ± 16.7 μmol TE/g
		Seeds (ethanolic extract)	Total polyphenols: 120.67 ± 4.38 mg GAE/mL Epicatechin: 63.36% ^3^ Gallic acid: 12.04% ^3^	DPPH: 69.55 ± 1.7 μg/mL (EC_50_) BCB: 7.07 ± 0.5 μmol Trolox/g ORAC: 514.76 ± 21.5 μmol TE/g
		Pulp (ethanolic extract)	Total polyphenols: 15.18 ± 0.79 mg GAE/mL Epicatechin: 39.01% ^3^ Gallic acid: 51.31% ^3^	DPPH: 792.43 ± 8.4 μg/mL (EC_50_) BCB: 0.74 ± 0.0 μmol Trolox/g ORAC: 102.82 ± 7.8 μmol TE/g
Cereja do Rio Grande (*Eugenia involucrata*)	[31]	Leaves (ethanolic extract)	Total polyphenols: 70.19 ± 1.88 mg GAE/mL Epicatechin: 49.43% ^3^	DPPH: 191.50 ± 0.3 μg/mL (EC_50_) BCB: 7.18 ± 0.6 μmol Trolox/g ORAC: 1393.3 ± 69.6 μmol TE/g
		Seeds (ethanolic extract)	Total polyphenols: 22.75 ± 0.50 mg GAE/mL Epicatechin: 33.52% ^3^ Gallic acid: 42.42% ^3^	DPPH: 346.70 ± 14.1 μg/mL (EC_50_) BCB: 3.38 ± 0.3 μmol Trolox/g ORAC: 168.66 ± 4.9 μmol TE/g
		Pulp (ethanolic extract)	Total polyphenols: 18.36 ± 0.66 mg GAE/mL Gallic acid: 24.55% ^3^	DPPH: 988.52 ± 22.4 μg/mL (EC_50_) BCB: 4.56 ± 0.1 μmol Trolox/g ORAC: 321.74 ± 9.8 μmol TE/g
Grumixama (*Eugenia brasiliensis*)	[31]	Leaves (ethanolic extract)	Total polyphenols: 73.25 ± 3.41 mg GAE/mL Epicatechin: 7.1% ^3^ Gallic acid: 69.4% ^3^	DPPH: 140.33 ± 3.7 μg/mL (EC_50_) BCB: 5.42 ± 0.4 μmol Trolox/g ORAC: 757.32 ± 34.7 μmol TE/g
		Seeds (ethanolic extract)	Total polyphenols: 42.60 ± 1.90 mg mg GAE/mL Epicatechin: 26.58% ^3^ Gallic acid: 45.56% ^3^	DPPH: 196.55 ± 3.9 μg/mL (EC_50_) BCB: 3.89 ± 0.1 μmol Trolox/g ORAC: 211.84 ± 9.3 μmol TE/g
		Pulp (ethanolic extract)	Total polyphenols: 26.69 ± 1.22 mg GAE/mL Epicatechin: 29.38% ^3^ Gallic acid: 9.02% ^3^	DPPH: 472.37 ± 3.2 μg/mL (EC_50_) BCB: 4.24 ± 0.1 μmol Trolox/g ORAC: 477.45 ± 23.3 μmol TE/g
Pêssego-do-mato (*Eugenia myrcianthes*)	[31]	Leaves (ethanolic extract)	Total polyphenols: 100.29 ± 3.29 mg GAE/mL Epicatechin: 0.76% ^3^ Gallic acid: 4.67% ^3^ Quercetin: 4.05% ^3^ Myricetin: 15.31% ^3^	DPPH: 83.80 ± 3.0 μg/mL (EC_50_) BCB: 5.27 ± 0.3 μmol Trolox/g ORAC: 644.31 ± 25.4 μmol TE/g
		Seeds (ethanolic extract)	Total polyphenols: 11.34 ± 0.49 mg GAE/mL Epicatechin: 14.23% ^3^ Gallic acid: 33.89% ^3^	DPPH: 734.51 ± 21.2 μg/mL (EC_50_) BCB: 1.13 ± 0.0 μmol Trolox/g ORAC: 96.24 ± 4.7 μmol TE/g
		Pulp (ethanolic extract)	Total polyphenols: 17.80 ± 0.89 mg GAE/mL Gallic acid: 6.68% ^3^	DPPH: 597.51 ± 9.5 μg/mL (EC_50_) BCB: 1.59 ± 0.1 μmol Trolox/g ORAC: 127.62 ± 5.8 μmol TE/g
Genipapo (*Genipa americana*)	[32]	Whole fruit (genipin-NADES extract)	Genipin: 11 ± 1% of extract	n.a.
Cutia fruit (*Carpotroche brasiliensis*)	[33]	Seeds (oil extract)	Hydnocarpate: 40.5% ^4^ Gorlate 16.1% ^4^ Chaulmoograte 14.0% ^4^ Hexadecanoate 6.0% ^4^ Octadecanoate 3.0% ^4^	n.a.
Macaúba (*Acrocomia aculeata*)	[45]	Pulp (whole matter)	Total polyphenols: 262.41 ± 7.63 mg GAE·100 g^−1^	β-carotene: 94.05 ± 0.05% protection ABTS: 735.35 ± 20.13 µMTrolox·g^−1^ fruit DPPH: 290.86 ± 7.68 g fruit·g^−1^ (IC_50_)
Blackberry jam fruit (*Randia formosa*)	[35]	Pulp (hydroalcoholic extract)	n.a.	EI: 6.78 ± 1.52 μA/V DPPH: 3.30 ± 0.23 μg/mL (IC_50_) ABTS: 18.17 ± 1.21 μg/mL (IC_50_) ↑ SOD: 11.99 ± 0.35 μg/mL (IC_50_) ↑ CAT: 69.53 ± 0.43 μg/mL (IC_50_)
		Seeds (hydroalcoholic extract)	n.a.	E: 2.52 ± 0.32 μA/V DPPH: 3.10 ± 0.10 μg/mL (IC_50_) ABTS: 15.93 ± 1.70 μg/mL (IC_50_)
Juçara (*Euterpe edulis*)	[46]	Pulp (freeze-dried methanolic extract)	Total polyphenols: 49.09 to 81.69 mg GAE/g d.b. ^5^	DPPH: 655.89 to 745.32 μmol TE/g d.b. ^5^ ORAC: 1088.10 to 2071.55 32 μmol TE/g d.b. ^5^
Cupuaçu (*Theobroma grandiflorum*)	[47]	Peel (aqueous extract)	Total phenolics: 200.67 ± 4.97 mg GAE/100 g DW Total flavonoids: 204.86 ± 9.57 mg QE/100 g DW	ABTS: 28.29 ± 1.98 µmol Trolox/g of sample DPPH: 93.79 ± 0.24 µmol Trolox/g of sample FRAP: 17.69 ± 0.38 µmol Trolox/g of sample ORAC: 17.27 ± 0.56 µmol Trolox/g of sample
		Peel (ethanolic extract)	Total phenolics: 263.55 ± 21.52 mg GAE/100 g DW Total flavonoids: 247.80 ± 7.30 QE/100 g DW	ABTS: 27.54 ± 1.75 µmol Trolox/g of sample DPPH: 64.25 ± 0.20 µmol Trolox/g of sample FRAP: 15.04 ± 0.74 µmol Trolox/g of sample ORAC: 16.81 ± 0.56 µmol Trolox/g of sample
		Seeds (aqueous extract)	Total phenolics: 788.94 ± 11.95 mg GAE/100 g DW Total flavonoids: 539.08 ± 28.63 QE/100 g DW	ABTS: 30.15 ± 1.81 µmol Trolox/g of sample DPPH: 93.58 ± 0.67 µmol Trolox/g of sample FRAP: 18.69 ± 0.72 µmol Trolox/g of sample ORAC: 15.08 ± 0.73 µmol Trolox/g of sample
		Seeds (ethanolic extract)	Total phenolics: 262.17 ± 0.77 mg GAE/100 g DW Total flavonoids: 505.87 ± 5.74 QE/100 g DW	ABTS: 38.09 ± 0.65 µmol Trolox/g of sample DPPH: 64.75 ± 0.63 µmol Trolox/g of sample FRAP: 18.95 ± 0.40 µmol Trolox/g of sample ORAC: 13.22 ± 0.44 µmol Trolox/g of sample
Pitanga (*Eugenia uniflora*)	[38]	Pulp (juice, 35%)	Anthocyanin: 119 µg/mL ^6^ Terpen: 30 µg/mL ^6^	n.a.
	[48]	Purple pulp (methanolic extract)	n.a.	DPPH: 3.1 ± 0.7 mmol trolox.100 g^−1^ FRAP: 3.1 ± 0.6 mmol trolox.100 g^−1^
		Red Pulp (methanolic extract)	n.a.	DPPH: 1.4 ± 0.1 mmol trolox.100 g^−1^ FRAP: 1.4 ± 0.3 mmol trolox.100 g^−1^
		Orange-fleshed pulp (methanolic extract)	n.a.	DPPH: 1.4 ± 0.0 mmol trolox.100 g^−1^ FRAP: 1.1 ± 0.1 mmol trolox.100 g^−1^

Captions: ^1^ Quantification. Units reported refer to experimental doses or extract concentrations; ^2^ Although IC_50_ determination was described, only percentage inhibition values at fixed concentrations were reported; ^3^ Percentage values refer to their relative peak area (%) among phenolic compounds identified by GC-MS; ^4^ Percentage values refer to the relative peak area (%) of fatty acid methyl esters identified by HRGC; ^5^ The values varied according to the maturation period; ^6^ Juice representative concentrations. The quantification of pitanga BC was based on a previous analytical study conducted by the same research group3.1. Metabolism and bioavailability.

## Data Availability

No new data were created or analyzed in this study. Data sharing is not applicable to this article.

## Abbreviations

The following abbreviations are used in this manuscript:

AA	Antioxidant activity
ABTS	2,2′-azino-bis(3-ethylbenzothiazoline-6-sulfonic acid
ACR	Acrolein
ALP	Alkaline phosphatase
ANT	Anthocyanins
ASC	Atypical Squamous Cells
BA	Benzoic acid
BCB	β-carotene bleaching
CAT	Catalase
CE	Catechin equivalents
CD45	Leukocyte common antigen
CGA	Chlorogenic acid
C3G	Cyanidin-3-glucoside
CNS	Central nervous system
CP	Protein carbonylation
CT	Condensed tannins
d.b.	Dry Basis
DCM	Dichloromethane fraction
DPPH	1,1-Diphenyl-2-picryl-hydrazyl
EI	Electrochemical Index
EPI	Epicatechin
FRAP	Ferric ion reducing antioxidant potential
GAE	Gallic acid equivalents
GFAP	Glial fibrillary acidic protein
GO	Glyoxal
GPx	Glutathione peroxidase
GSH	Reduced glutathione
HFD	High-fat diet
HFG-1	Human gingival fibroblasts
IL	Interleukin
IFN-γ	Interferon gama
LPO	Lipid peroxidation
LPS	Lipopolysaccharide
MeHg	Methylmercury
MGO	Methylglyoxal
MPO	Myeloperoxidase
MCP-1	Monocyte chemoattractant protein
MDA	Malondialdehyde
MUFAs	Monounsaturated fatty acids
n.a.	Not assessed
NF-κB	Nuclear factor kappa B
NO	Nitric oxide
NT	Nitrite
ONOO	Peroxynitrite
ORAC	Oxygen Radical Absorbance Capacity
PAR	protease-activated receptor
PUFAs	Polyunsaturated fatty acids
PM	Phosphomolybdenum
QE	Quercetin equivalent
RAS	Renin–Angiotensin System
RNS	Reactive Nitrogen Species
ROS	Reactive oxygen species
SOD	Superoxide dismutase
TAE	Tanic acid equivalent
TBARS	Thiobarbituric acid reactive substances
TE	Trolox equivalent
TNF-α	Tumor Necrosis Factor Alpha
UFAs	Unsaturated fatty acids
±	Standard deviation
